# Nanovectorization of Prostate Cancer Treatment Strategies: A New Approach to Improved Outcomes

**DOI:** 10.3390/pharmaceutics13050591

**Published:** 2021-04-21

**Authors:** Kenneth Omabe, Clément Paris, François Lannes, David Taïeb, Palma Rocchi

**Affiliations:** 1 Centre de Recherche en Cancérologie de Marseille, CRCM, Inserm UMR1068, CNRS UMR7258, Aix-Marseille University U105, Institut Paoli-Calmettes, 13273 Marseille, France; kenneth.omabe@inserm.fr (K.O.); clement.paris@inserm.fr (C.P.); francois.lannes@ap-hm.fr (F.L.); david.taieb@ap-hm.fr (D.T.); 2 Department of Biochemistry & Molecular Biology, Alex Ekwueme Federal University, Ndufu-Alike Ikwo, PMB 1010, Abakaliki 84001, Nigeria; 3 Biophysics and Nuclear Medicine, La Timone University Hospital, European Center for Research in Medical Imaging, Aix-Marseille University, 13005 Marseille, France

**Keywords:** prostate cancer, CRPC, Non-AR therapeutic targets, nanotherapies, nanotheranostics

## Abstract

Prostate cancer (PC) is the most frequent male cancer in the Western world. Progression to Castration Resistant Prostate Cancer (CRPC) is a known consequence of androgen withdrawal therapy, making CRPC an end-stage disease. Combination of cytotoxic drugs and hormonal therapy/or genotherapy is a recognized modality for the treatment of advanced PC. However, this strategy is limited by poor bio-accessibility of the chemotherapy to tumor sites, resulting in an increased rate of collateral toxicity and incidence of multidrug resistance (MDR). Nanovectorization of these strategies has evolved to an effective approach to efficacious therapeutic outcomes. It offers the possibility to consolidate their antitumor activity through enhanced specific and less toxic active or passive targeting mechanisms, as well as enabling diagnostic imaging through theranostics. While studies on nanomedicine are common in other cancer types, only a few have focused on prostate cancer. This review provides an in-depth knowledge of the principles of nanotherapeutics and nanotheranostics, and how the application of this rapidly evolving technology can clinically impact CRPC treatment. With particular reference to respective nanovectors, we draw clinical and preclinical evidence, demonstrating the potentials and prospects of homing nanovectorization into CRPC treatment strategies.

## 1. Introduction

### 1.1. Prostate Cancer and CRPC Emergence

Prostate cancer (PC) is the most frequent male cancer in the developed world. The majority of the localized PCs can be treated with surgery or radiation (Table 1). However, if the disease is diagnosed at the extra-prostatic or metastatic stage, neither radiation nor surgery can offer a good clinical benefit. While Androgen Deprivation Therapy (ADT)/castration represents a consensus treatment for advanced PC, it has become more clear that this disease does not uniformly and completely regress following ADT [1] and may account for the short-lived clinical benefit of 2–3 years [2,3,4]. During this period, most patients become unresponsive to ADT and progress to ADT-resistant PC, a state that is termed Castration-Resistant Prostate Cancer (CRPC) [5,6]. Unfortunately, to date, this phenotype is virtually untreatable and ultimately, patients of this category usually die of the disease. As a sequel to its bad prognosis, CRPC has remained a serious challenge to both clinicians and drug developers.

### 1.2. Classical Androgen Receptor Pathway in CRPC Progression

Androgens are the primary regulators of PC cell growth and proliferation. Blocking the synthesis of androgen and/or androgen receptor (AR) signaling is the gold standard in the treatment of metastatic PC and can be achieved through surgical or medical castration. Nonetheless, most tumors first respond to ADT, while others become resistant to therapy within two years [7]. Studies show that numerous factors contribute to AR reactivation, despite castrate serum levels of androgens thereby causing PC recurrence. These include changes in AR expression and structure through gene amplification, mutation, and alternative splicing. Changes in steroid metabolism, cell signaling, and coregulatory proteins are also important contributors to AR reactivation in CRPC [8]. However, emergence of CRPC has been linked to prolonged inhibition of the androgen receptor-signaling pathway, giving rise to androgen receptor-independent clonal evolution [9].

### 1.3. Non-Androgen Receptor Pathways in CRPC Progression

While the androgen receptor (AR) signaling pathway plays an important role in the emergence of CRPC and has been well recognized as a hallmark of CRPC [10], recent findings have identified and characterized several non-AR related pathways and targets that drive PC progression independent of AR-axis. Following this discovery, there has been continued interests in targeting alternative pathways that contribute to PC progression and resistance to therapy such as stress response and cell survival pathways (e.g, targeting Hsp27 [11] and TCTP [12] in CRPC). Others include Tumor microenvironment, Microtubules, angiogenesis, intracellular signal transductions, DNA damage response, anti-apoptotic proteins bcl-2 [13], gene fusion [14], deregulation of tumor suppressor genes [15], alternative splicing phenomenon [16], miRNA-dependent post-transcriptional modification and epigenetic alterations [17]. As a result, several experimental and approved therapies targeting pathways and targets indifferent of AR have been developed for PC treatment [1] (Table 2) (http://clinicaltrials.gov (accessed on 10 February 2021).

**Table 1:** Different treatment options for prostate cancer and their pitfalls.

Treatment	Strenghts	Limitations	Ref
SURGERY	Effective for localized tumours, Combine with pre/postoperative chemo/radiotherapies	Ineffective for metastatic PC Recurrence rate is high	[18]
RADIATION THERAPY	Effective with organ specific tumor. Prevents post-operative reoccurrence	Synonymous with high rate of collateral lethality	[19]
(HORMONAL THERAPY)ADT	Effective for advanced cancers	High rate of recurrence and emergence of CRPC	[20]
CHEMOTHERAPY	Effective in combination with ADT	Synonymous with with high rate of collateral lethality	[21]
GENOTHERAPY	Inhibits specific genes that drive Prostate Cancer. More effective in combination with chemotherapy	Ineffective as a monotherapy	[22]

### 1.4. Current Prostate Cancer Treatment Strategies and Their Limiting Factors

As highlighted in Table 1, many treatment strategies have evolved for PC treatment over the years. However, combination of any of the treatment options with chemotherapy has been significantly more efficacious than monotherapies [23]. As a result, the last few years has witnessed a significant increase in the number of FDA-approved chemotherapy for PC treatment. No doubt that the therapeutic potentials of these agents are incontestable. However, limitations are common that hinder their clinical success: either inherent in the drugs themselves (pharmacodynamics) or encountered during their pharmacological journeys in patients (pharmacokinetics).

Firstly, a plethora of biological barriers exist to the detriment of the efficacy of the cancer drugs. Consequently, there is increased incidence of non-specific distribution en route the tissue or cellular compartments of therapeutic interest. These barriers could range from penetration of cellular membranes, humoral attacks, efflux pump dependent eviction to endosomal entrapment [24]. In the end, only about 1 in 10,000 drug molecules would be able to make it to the target site, thus giving rise to poor efficacy [25]. In order to step up bioavailability and in turn, a relatively adequate amount of drug at the disease site to achieve desired effects, much higher doses are administered, resulting in an increased rate of collateral toxicity and incidence of multidrug resistance (MDR) [26].

More specifically, the physicochemical property of Paclitaxel essentially limits its administration in molecular form. Its poor solubility value (0.0015 mg/mL) negatively affects their polycyclic chemistry in aqueous solution [27,28] and renders it inappropriate for intravenous injections [29].

Recently, combination therapy involving the use of cytotoxic agents and antiandrogen regimens has emerged as a formidable strategy to combat CRPC. Docetaxel, in combination with Prednisone, is associated with improved clinical outcomes [30,31]. Docetaxel inhibit the depolymerization of the mitotic spindles to block cell replication. However, its administration is characterized with a high grade toxicity affecting rapidly dividing cells such as the bone marrow, hair follicles, germ and blood cells, etc. [32,33]. Major side effects include neutropenia, hypersensitivity reactions, stomatitis, peripheral neuropathy, and fluid retention [34]. Even though, with the advent of premedication regimens and longer administration schemes, the hypersensitivity reaction associated with paclitaxel or docetaxel has been fairly reduced [35]. However, recent reports have implicated docetaxel in fatal interstitial pneumonitis in CRPC patients [36]. In fact, death due to docetaxel-induced toxicity accounts for about 2% of the population in a 2045 patient’s study [37].

In addition, co-delivery of multiple agents at the same time is apparently an attractive strategy in CRPC therapy. However, this approach is limited by the variations in the independent pharmacokinetics, bio-distribution, and clearance rate of the respective agents, which makes it difficult for the individual efficacies of these agents to work in an operational consonance, thereby upturning the essence of synergism.

For these reasons, there is a high demand for a drug delivery system that would pharmacologically guarantee improved stability, solubility, safety and specificity of a variety of chemotherapeutic agents. One way to realize this goal is through the field of nanotherapeutics [38], which allows nanovectorization of drug agents in delivery platforms that would promote targeted delivery (passive and active targeting) with minimal toxicity and an improved therapeutic index.

### 1.5. Nanoparticles in Prostate Cancer Therapies: The Awaiting Possibilities

Given the challenges of collateral toxicity and non-specific distribution of PC therapies, arising from convectional delivery methods, which translates to poor efficacy, scientists have embarked on the search for a veritable alternative in order to contend with these challenges. Nanotechnology provides the platform with inherent characteristics to guarantee the safety, specificity and therapeutic efficacy of advanced prostate cancer therapies. These nanoparticles consist of biodevices and materials with functional ductility and various structural characteristics such as polymers, lipids, inorganic carriers and biological scaffolds to create nanoscale drug carrier systems (nanoparticles) capable of specific delivery of cancer therapeutics [39].

Indeed, with the advent of nanovectors and nanovectorization of PC therapies, it is possible to [1] deliver a high dose of anticancer agents, [2] co-deliver two or more therapeutic molecules in a single nanoformulation, [3] achieve a payload delivery of drug agent, [4]) reduce toxicity and [5] improve therapeutic outcome.

For instance, functionalized nanovectors can consolidate the individual pharmacokinetics and pharmacodynamics of drug agents into one vehicle and increase the likelihood of delivering each agent to the tumor cells at a ratiometric dose [40]. Additionally, we and others have recently demonstrated the possibility to co-deliver a Chemogene (chemo-and-gene based therapy) in a single nanoconstruct to synergize gene silencing and cytotoxicity for CRPC therapy [41,42,43]. Indeed, nanoparticles represent an excellent drug delivery system with enhanced targeted drug delivery capabilities via the passive or the active mechanisms. They have shown to decrease drug toxicity, concentrate drug at disease sites, prolong the systemic circulation of the drug as well as protect drugs from humoral attacks [44]. While prostate cancer therapeutics has not enjoyed sufficient attention in the field of nanomedicine, available data indicate a promising future. For example, near-infrared fluorescence (NIRF) imaging of PC-3 xenograft-bearing mice showed that PEG-micelles were selectively accumulated at the tumor site with minimal distribution in major organs, including liver and spleen [45,46]. Similarly, delivery of paclitaxel via PEG_5K_-embelin_2_ micelles leads to superior antitumor activity compared to Taxol in murine models of breast and prostate cancers [46]. Xang and colleagues have reported the impact of oxygenation induced by per-fluoro carbon nanodroplet on accumulation in prostate tumors xenograft. They observed a particle accumulation in mice tumor within 24 h, with a reduction of the tumor hypoxia without enhancing oxygen breathing [47]. With these available testimonies and more on the promises of nanoparticles in CRPC treatment, it is sufficiently acceptable to assert that nanovectorization posits to revolutionize the treatment of CRPC.

### 1.6. Classification of Therapeutic Nanoparticles in Prostate Cancer

Ideally, for a nanovector to be qualified as a drug delivery material, it must be non-toxic, biocompatible, non-immunogenic, biodegradable, possess the ability to avoid the Reticulo-endothelial system (RES) and renal clearance systems [48,49]. These factors are particularly important to ensure that the perceived gains associated with nanovectorization of drugs are effectively maximized.

**Figure 1:**
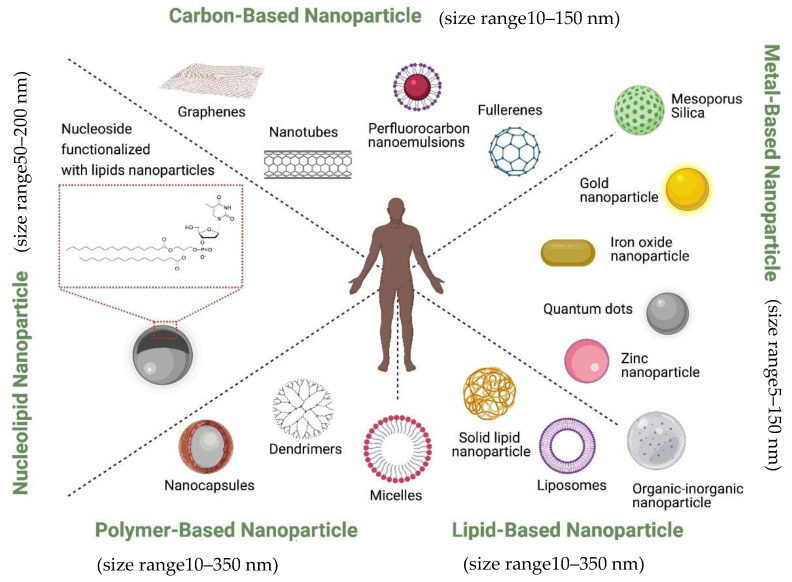
Schematic illustration showing the nanoparticles classified according to their chemical composition (created with Biorender).

Currently, nanoparticles are classified according to their chemical compositions (Figure 1): (1) metal-based nanoparticles to include quantum dots, iron oxide and gold nanoparticles, zinc nanoparticle, mesoporous silica, and organic-inorganic nanoparticles [50], (2) carbon-based nanoparticles such as nanotubes or fullerenes [51], (3) polymer nanoparticles such as Nanocapsules or dendrimers [52,53], (4) lipid-based nanoparticles including liposomes and solid lipid nanoparticles [54,55], and (5) a new class based on nucleolipid nanoparticles.

They can also be decorated with functional moieties such as specific ligands to induce active targeting. It is also possible to further innovatively engineer them to bring together the active and passive targeting mechanisms such as, EPR effect, RES avoidance, bio-recognition moieties, membrane trafficking and efficient intracellular delivery, remote drug activation and controlled drug release to act in efficacious operational harmony. Ferrari and colleagues described this group of nanovectors as ‘Logic embedded Vectors’ (LEV) and rationalized their potential in personalized medicine [39].

These nanoparticles are designed to take advantage of the exclusive tumor signatures such as Enhanced Permeability and Retention (EPR) phenomenon, pH, hypoxia, as well as overexpression of tumor-specific receptors [56,57,58] in order to selectively home into tumor cells with minimal/no effect on their normal counterparts.

At this moment, several nanovectorized drugs have received FDA’s approval while some are at different phases of clinical or preclinical development [59,60,61,62]. While the field of nanotherapeutics has been substantially studied, developed and utilized in the treatment of various cancer types, its enormous potentials have been underutilized in prostate cancer therapy, both in preclinical and clinical settings. Here, we review the various conventionally used nanoscale drug carriers such as liposomes, micelles and dendrimer nanovectors. We bring a deep insight into their structural designs and mechanisms of action. Available knowledge on their applications in delivering cancer chemotherapeutics is provided with new specific possibilities in transforming prostate cancer treatment strategies.

## 2. EPR Effect and Active Targeting in CRPC Therapy

**Figure 2:**
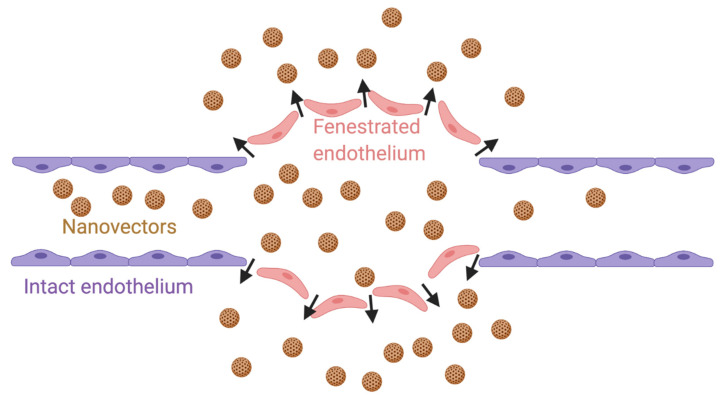
EPR effect. Nanovectors equipped with hydrophilic and flexible polymers escape opsonization and are able to diffuse selectively through the tumor vascular endothelium (orange color) but not through the endothelium of healthy tissue (blue color). Created with Biorender; Adapted from [68], Collège de France, 2013.

Enhanced Permeability and Retention (EPR) Effect: Tumor cells, in a frantic attempt to mitigate the circumstance of limited diffusion, which negatively influences nutrient supply, oxygen supply and waste removal, engage in neoangiogenesis to step up vasculature. In order to avoid the imminent consequences of hypoxia, ischemia and toxicity, this process seems to occur very quickly and as a result, the contributions of the angiogenic regulators are largely excluded. This gives rise to highly disorganized and fenestrated tumor vessels, with discontinued endothelial linings and undesirable permeability to particles of up to 700 nm in diameter [63,64]. This pathologically orchestrated phenomenon is referred to as enhanced permeability and retention (EPR) effect [65] (Figure 2). Maeda et al. conceptualized and clearly demonstrated how angiogenic flaws associated with rapid tumor growth promoted site-specific accumulation and retention of nanoparticle-drug conjugates at the tumor site [65,66]. Taking advantage of the pressure gradient and leaky vessels at the tumor site, the nanoparticle-based drug accumulates at the tumor site, extravasates into the tumor microenvironment, and prolongs the release of therapeutic agents within the tumor with a resultant improvement in the treatment outcome. This emerged a land slide achievement in the field of nanomedicine and widened the scope of nanoparticle-based drug delivery via passive targeting such that new nanodrug formulations received approval for the treatment of other disease conditions such as fungal infection, hepatitis A, multiple sclerosis and end stage renal disease [67]. However, in order to maximize the advantages offered by EPR in the passive delivery of drugs, the size and surface properties of the nanocarrier must be controlled to avoid uptake by the reticuloendothelial system (RES) as previously described. The ideal size range to benefit from the EPR effect is between 10 to 200 nm as particles that are too small would be cleared by the kidneys, preventing accumulation into the tumor site, and particles that are too large will not adequately penetrate the tumor vasculature and interstitial space.

Active Targeting Mechanisms: Conversely, the nanovectors can be further engineered to improve their delivery efficiency via the active targeting strategy. This approach has been proven to be very specific and effective in delivering drug agents for the treatment of many disease conditions, including cancer. This strategy is defined by decorating the outer surface of the nanovectors with bio-recognition moieties such as antibodies, ligands, aptamers, small peptides to specifically target a tumor-specific receptor/ligand overexpressed on the tumor or tumor vasculature [69]. For instance, an antibody directed against the transmembrane receptor (CD33) specific on myeloid lineage, was immune-conjugated with Calicheamicin for the treatment of Acute Myelogenous Leukemia [70]. By way of ligand-receptor interaction, the anti-tumor agent is internalized into the tumor cells expressing this receptor via receptor-mediated endocytosis which is very critical for optimal targeted delivery of therapies [71,72]. However, it appears literally not feasible for a particular receptor to be exclusively expressed by only tumor cells. As such, overexpression of these receptors represents tumor specific signature that can be targeted in this fashion [73]. A further classic example is the development of therapeutic monoclonal antibodies to target membrane receptors that are reservedly overexpressed on tumor cells. This was demonstrated in the use of Herceptin (Trastuzumab) to target HER2 receptor overexpressed in a sub-type of breast cancer [74]. Furthermore, conjugation of a nanovector with cyclic RGD peptide to target neovasculature markers such as alpha and Beta integrins [75], VEGF or anti-VEGFR to target vesicular endothelial growth Factor receptor (VEGFR) [76,77], or development of antibody or ligand to target the prostate specific membrane antigen (PSMA) [78,79] constitute active drug targeting strategies that have been well developed and utilized. Recently, some reports have described the use of PSMA ligand for active targeting. Felber et al. described a new coating ligand for AuNPs, allowing direct labeling with radionuclide ^99m^Tc functionalized with a small prostate specific membrane antigen (PSMA). Bio-distribution assay revealed higher stability and significantly higher uptake for particles greater than 14 nm [80,81]. Yari et al. also reported the development of a prostate specific membrane antigen (PSMA)-tagged liposome for specific targeting of advanced prostate cancer tumoral cells. Their results have shown the efficiency of active targeting of prostate cancer cells with PSMA ligand [82].

## 3. Some Nanoparticles and Their Clinical Adaptations to CRPC Treatment

While only a few works on nanovectorization-based therapies have progressed to clinical studies for the treatment of prostate cancer, published articles lending support to the concept of nanovectorization-based drug delivery for prostate cancer therapy have grown over the past decade. Table 3 contains a list of recent articles describing the development of nanoparticle for prostate cancer treatment (Table 3).

**Figure 3:**
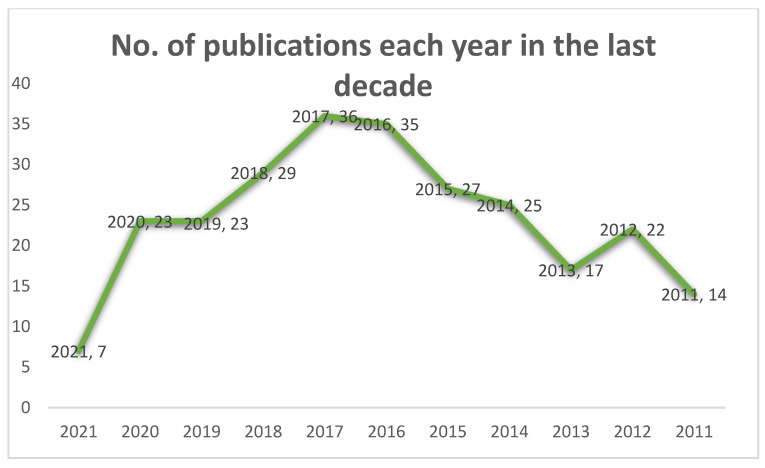
A graphical representation of the number of published articles relating to prostate cancer and nanoparticles drug delivery between 2011 and 2021.

However, we recently carried out an advanced search for publications in this field: ‘Prostate cancer and nanoparticles drug delivery’ on the PubMed Data base to understand how research in this field has progressed in the past 10 (2011–2021) years. We found 232 publications, which increased to 605 when ‘drug delivery’ was omitted in the search field out of 41,295 publications on prostate cancer itself within the same period. This data is represented graphically in Figure 3 below (Figure 3). The highest number of publications in this field according to our search filters was in 2017, which suggest that, indeed, research in the nanovectorization-based therapies for prostate cancer treatment is increasingly growing. Table 4 contains some nanoformulation-based drugs in clinical trials for prostate cancer treatment (Table 4).

The majority of these articles highlights the gains and drawbacks, the anatomical and physiological advantages associated with nanoparticles—aided delivery of PC treatment strategies such as chemotherapy, Genotherapy, antitumor peptides, etc., thereby improving their efficacy and clinical outcome. As of today, liposomes, micelles, polymer conjugates and dendrimers are particularly useful and fundamental in the development of drug delivery platforms. They possess attractive biological properties that make them biocompatible, biodegradable, non-toxic, ability to encapsulate both hydrophobic and hydrophilic drug agents and protect drugs from systemic inactivation. They encapsulate chemotherapeutic agents, Genotherapies (siRNA or DNAs) and small molecule drugs and localize them at tumor sites. They can be used as unmodified (passive delivery) or modified with bio-recognition moieties (active mechanism) and are discussed below.

### 3.1. Liposomal Nanoparticles

**Figure 4:**
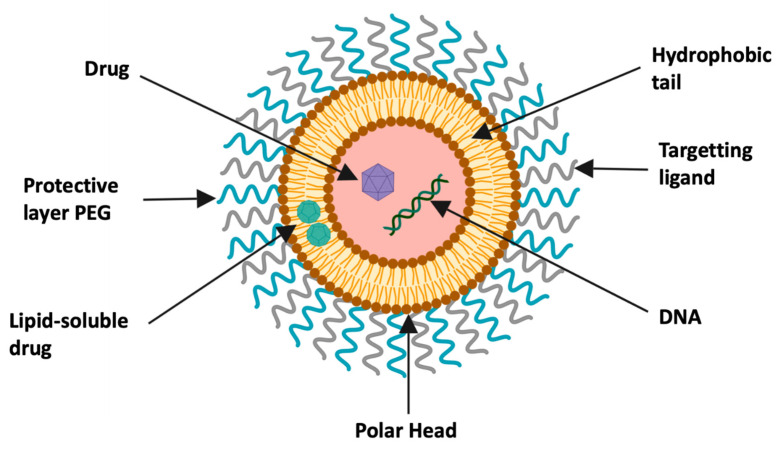
Schematization of a liposome used as nanovector. Liposomes generally consist of a lipid bilayer (phospholipids and cholesterol). The drug is encapsulated in the hydrophobic central region. The outer surface of the vector may contain polyethylene glycol (PEG) and ligands, created with Biorender [94].

Liposomal nanoparticles are small artificial, self-assembled vesicular nanostructures derived from phospholipids and cholesterol molecules. They represent a very good membrane models and are widely used for studying membrane fluidity. However, liposomes have been recently found to be extremely useful in nanotherapeutics for targeted delivery of pharmacological agents such as proteins, Oligonucleotides (ASO), siRNA or chemotherapeutic drugs [55,93]. If the drug is hydrophobic, the encapsulation is within the central cavity, but in the case of rather neutral or hydrophilic drug, it will then be loaded on the surface of the lipid membrane [55] (Figure 4).

The first liposome-based drug used in the clinic was the non-PEGylated liposome that encapsulates Doxorubicin (marketed as Myocet^TM^ in the USA), which increased the elimination half-life of the encapsulated doxorubicin in the blood compared to free doxorubicin (DOX) [95]. Upon intravenous administration of Myocet^TM^, it is internalized and subjected to enzymatic degradation of the lipid bilayer, which consequently releases the drug (doxorubicin in this context). This drug then diffuses actively or passively to the target tissue [62]. Many more of such formulations are in different developmental stages such as liposomal Vincristine for the treatment of acute lymphoblastic leukaemia [60,96].

To further fortify liposomal drug formulation and enhance its hydrophilicity and protection from the RES, surface modification with a hydrophilic polymer; polyethylene glycol (PEG) was used to realize PEGylated liposomal doxorubicin (marketed as Doxil^®^ in the USA and as Caelyx outside USA). PEG has been shown to prevent opsonization and recognition by macrophages and enable the liposome to maintain a longer circulation time in the blood to accumulate at tumor sites, aided by the EPR effect. Within the tumor environment, the liposomal content (in this case, antitumor agent) can be released near the tumor particularly due to exclusive pH variation inherent within the tumor territory, resulting in the burst of the lipid bilayer. Experimental validations have proved that PEGylation of liposomal doxorubicin (Doxil^®^) prevented RES sequestration, drug inactivation and significantly increased circulation time from 2.5 h to 55 h as well as decreased clinical cardiotoxicity in solid tumor patients compare to both DOX and Myocet^TM^ administration [93]. As a result, the US Food and Drug Administration (US-FDA), for the first time, granted clinical approval to Doxil^®^ in 1995 for the treatment of Kaposi’s Sarcoma [62,97] and subsequently for the treatment of recurrent breast and ovarian cancers [98,99,100]. In fact, following this milestone achievement, the last two decades have witnessed a dramatic rise in the development of several liposome-based drugs for cancer treatment with many of them at the preclinical stage, while others are being assessed at different stages of clinical development such as PEGylated liposomal daunorubicin (DaunoXome^®^), Vincristine (Onco-TCS) or PEGylated liposomal cisplatin (SPI-77) [101,102].

Specifically, and as highlighted above, docetaxel, in combination with prednisone, appear to be the standard of care for CRPC [30,31] with high anticancer activity [103], but highly toxic to normal cells [104]. Given the advances and prospects of liposomal drug technology, it is believed that liposomal intervention into the use of docetaxel in CRPC treatment would avail patients with a much longer survival benefit than earlier cited years [2,3,4]. Studies have demonstrated improved efficacy of liposomal drugs in prostate cancer models. For example, the combined effect of liposomal forms of curcumin and resveratrol more significantly inhibited tumor growth and induced apoptosis in PTEN-CaP8 cell lines with a concomitant reduction in prostatic adeno carcinoma in vivo in PTEN mice [105]. Additionally, curcumin-loaded liposomes decorated with PSMA antibodies tested in LNCaP and C4-2B efficiently inhibited cellular proliferation at a very low dose (5–10 µM) compare to free curcumin [106]. In the wake of these potentials, it is regrettable to note that only very few preclinical studies on liposomal docetaxel have focused on prostate cancer. This is even more worrisome given the impact of liposomal formulations on other antitumor agents such as gemcitabine [107], paclitaxel [108], Mitoxantrone [109] and doxorubicin [110,111,112,113] on prostate tumors.

Furthermore, a cohort study on several advanced tumors revealed a high incidence of liposomal docetaxel tolerance at elevated doses [114]. Nevertheless, contrasting results had surfaced when liposomal doxorubicin was investigated on prostate tumor xenograft. Three different studies realized similar outcome of significant tumor inhibition [115,116]. Another study involving liposomal gemcitabine in an LNCaP model of prostate cancer xenograft tumors showed more than a 40-fold potency in antitumor activity relative to the free counterpart [117].

#### 3.1.1. Liposomal Nanoparticle in Therapeutic Gene Delivery

Antisense Oligonucleotide (ASO) therapy is increasingly taking a center stage in fighting drug resistance in cancer treatment through inhibition of genes implicated in drug resistance. However, poor cellular uptake and inefficient intracellular delivery of ASO remains a serious concern to ASO strategy. Liposomal encapsulation of ASO appears promissory to remediate this challenge. Liposomal ASO has been shown to be effective in inhibiting multi-drug resistant, mice-bearing tumors xenografts of ovarian cancer. Co-encapsulation of ASO and DOX in a liposome nanoformulation essentially improved the antitumor activity [118].

Other Liposome-based enhanced gene delivery approach includes the targeting of the angiogenic gene (VEGF), which is overtly overexpressed in many cancer types with small interfering RNA duplexes (siRNA) loaded in Chitosan-coated liposomal formulations and has been found to exhibit an efficient gene silencing activity in breast cancer cells lines [119]

#### 3.1.2. Liposomal Drug Loading and Release

Drug loading into the aqueous core of the liposome is made possible through a remote loading protocol like pH gradient and ammonium sulphate methods for doxorubicin and vincristine, respectively [120,121]. Liposome nanovectors can be designed to release their drug conjugates inside the endosomes or lysosomes displaying acidic pH and high enzymatic content in order to control the pH–mediated release into subcellular therapeutic targets.

As of now, with the trajectory of preclinical and clinical successes associated with the liposomal drugs in cancer treatment, it is sufficiently acceptable to assert that liposomal encapsulation holds a very promising future for CRPC with an abundance of economic opportunities for drug developers to exploit

### 3.2. Micellar Nanoparticles

**Figure 5:**
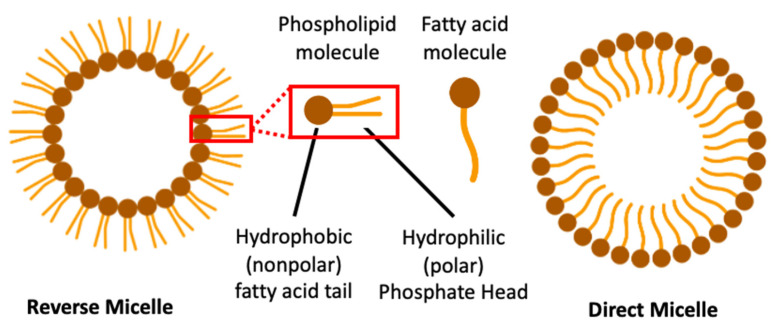
Showing the reverse and direct micellar structures. Micelle nanovectors consist of direct micelles with the anti-cancer agent positioned either on the surface for hydrophilic drugs (e.g., antisense oligonucleotide for example) or inside for hydrophobic drugs (e.g., chemotherapeutic agents). (Created with Biorender).

Micellar nanoparticles are spheroidal nanoscale systems that have been useful in the targeted delivery of anticancer drugs. They are formed by self-assembly of an amphiphilic block of copolymers in an aqueous medium. This spontaneous auto-aggregation, which occurs under certain high concentrations (critical micellar concentration: CMC), gives rise to a nano-dimensioned molecular structure having a hydrophilic polar head and a hydrophobic chain all oriented in the same direction with a molecular size of about 1 to 300 nm. Micelles are in two forms: direct and reverse micelles. Direct micelles have their hydrophobic nonpolar chains inside the solvent while their hydrophilic polar heads interact with solvent at the surface. However, the hydrophilic polar heads of the reverse micelles are found facing inward while the non-polar hydrophobic chains are positioned outwardly [122] (Figure 5). The micellar hydrophobic core lends itself well to encapsulation of poorly soluble anti-cancer drugs such as Paclitaxel (an effective anti-cancer agent inhibiting tumor cell microtubule growth) and enhances its solubility in biological medium.

The micelles conventionally used as nanovectors are the direct forms of micelles with a size generally ranging between 10 and 100 nm. They can be enhanced by conjugating hydrophilic polymers such as poly (ethylene glycol) (PEG), tagged with a hydrophobic polymer (Polymeric Micelle: PM). Commonly used hydrophobic polymers for micellar enhancement include poly (propylene oxide) (PPO), poly (D, L-lactic acid) (PDLLA), poly (ε-caprolactone)) (PCL) or poly (L-aspartic acid) (PLAA), biodegradable polyesters and polyorthoesters, phospholipids or long chain fatty acids. These block copolymers undergo auto-assemblage to form a hydrophobic core bordered by a hydrophilic corona [123,124]. The combined physicochemical properties of these copolymers confer amphiphilic characteristics on the PM, thereby rendering them excellent candidates for intravenous administration of a variety of chemotherapeutics such as antitumor agents. PEG is the most widely used hydrophilic polymer in targeted drug delivery even though other polymers such as poly(N-vinyl pyrrolidone) (PVP) or Poly(N-isopropylacrilamide) (pNIPAM) are also good water-loving polymers [125,126]. Being non-toxic with unique physicochemical properties, it offers huge advantages to a wide range of drugs and drug conjugates such as proteins, enzymes, small molecules, liposomes and inorganic nanoparticles [127,128]. It does not only increase the drug-loading capacity of the micelles, but also prolongs drug retention time, protects drugs from humoral attacks, and as a result, improves their therapeutic potentials.

Drug-PM conjugates are structured through physicochemical and electrostatic interactions and are compatible with both active and passive mechanisms of tumor targeting. These conjugates can be further fortified to enhance drug solubility in water to as much as 9000 times, the solubility of an apparently soluble drug [129,130].

#### 3.2.1. Polymeric Micelles in Targeted Delivery

Currently, polymeric micelles are being tried in various phases of clinical trials with promising outcomes. For example, impressive antitumor efficacy was preclinically evident with doxorubicin encapsulated with PEG–PLA(polyactide)A-micelles conjugate. This nanoconstruct, which is also referred to as NK911, prolonged the systemic circulation time and nearly tripled the half-life of the encapsulated drug with decreased drug clearance [131]. For the treatment of refractory malignancies such as CRPC, paclitaxel was first formulated with PM-Micelle (genexol-PM: PEG- PDLLA-Paclitaxel: Poly(ethylene glycol) methyl ether-block-poly(D,L lactide) and was tested in clinical trial. Results from this trial have shown that genexol-PM offers much more tolerable clinical reactions compared to the convectional formulation of paclitaxel that contains Cremephor^®^ EL, which has been associated with the occurrence of hypersensitivity reactions in patients [130]. Following these findings, elevated doses of paclitaxel allowed into genexol-PM resulted to a more efficacious antitumor activity of paclitaxel in patients who previously showed resistance to conventionally administered paclitaxel [129]. These complex micellar-paclitaxel formulation, indeed, significantly increased its solubility from 0.0015 mg/mL to 2 mg/mL and apparently allows the drug to evade rapid phagocytosis by the Reticuloendothelial System (RES) preventing recognition by opsonins [129,130].

While polymeric micelles appear to be clinically relevant through the passive targeting mechanism, there is a huge possibility for improved delivery through the active targeting strategy. While the passive EPR effect only facilitates efficient localization of PMs into the tumor interstitium, active targeting promotes their uptake and internalization via a receptor mediated endocytosis [132]. Indeed, EPR has been substantially utilised in drug delivery, albeit, its mechanisms are not without limitations as they lack molecular specificity. Notwithstanding, the use of a receptor-based active targeting strategy surpasses the shortfalls associated with EPR. Very significantly, combination of EPR effect with active targeting would be of great value in achieving the desired and impactful treatment. PM-based nanovectorization of anti-tumor agents that is coupled with surface fortification with targeting entities (ligands or antibodies) that specifically and selectively interact with a tumor-specific receptor underscores the concept of PM—active targeting. Upon this interaction, the PM-drug-ligand complex is internalized into the tumor cells via a receptor-mediated endocytosis, followed by sub-cellular trafficking and release stimulations [133]. In this way, high bioavailability, low systemic toxicity and improved therapeutic outcome are achieved. Intriguing reports have shown insignificant disparity in the way the drug is accumulated to tumor sites between the ligand targeted and non-ligand targeted nanovectors [134]. This was perceived to be due to the fact that both carriers leverage on the benefits of the pressure gradient favoring the tumor interstitium offered by EPR. Until the nanovectors extravasate into the tumor microenvironment, ligand conjugation to nanovector appears inconsequential. However, extravasation of the ligand-tagged nanovectors may become unnecessary if; (1)the targeted tumor receptor is resident on the tumor endothelial cells and not on the tumor cells; (2)the targeted tumor cells are situated in the vascular compartment; (3) the target tissue has high accessibility of the vasculature such as alpha or beta Integrin, ligand or antibody targeting VEGFR or the prostate specific membrane antigen (PSMA) [131]

#### 3.2.2. Polymeric Micelle Drug Release

At the tumor location, the molecules must be released from the micelles in order to achieve cytotoxicity. Different approaches of drug release stimulation such as temperature, PH or ultrasound have been studied for this purpose. For example, pH stimulation is based on the fact that tumoral tissues tend to have abnormally acidic pH (up to 5.7) compared to normal tissues (7.4) because of their glycolytic metabolism [134]. Therefore, the release can be triggered by developing labile acid bonds or by using selective protonations of pH-sensitive compounds inside the membranes. Similarly, studies have also been conducted to develop magnetic resonance imaging (MRI), computed tomography (CT) and positron emission tomography (PET) trackable micelles [128]. Thus, multiple functional components can be incorporated into a single micelle to combine tumor targeting and imaging [135,136].

Another mechanism of drug release currently being developed combines imaging and ultrasound release stimulation of anti-cancer agents [135,137]. The tumoral irradiation makes it possible to stimulate drugs release from the micelles and to temporarily alter targeted cell membranes, thus increasing the quantity of drugs ingested. In addition, ultrasound promotes the drugs diffusion and infiltration into tumors. Using ultrasound for tumor imaging and treatment appears particularly interesting because of the simple implementation and its low cost.

**Figure 6:**
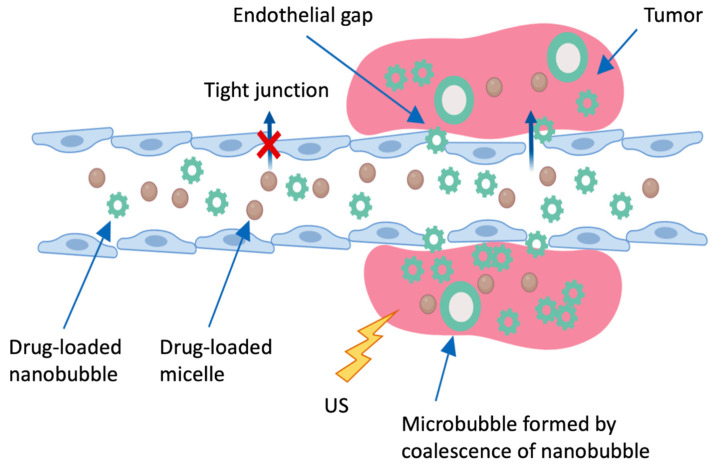
Schematic representation of the targeting of anti-cancer agents through defective tumor microvasculature using the system of delivery of echogenic particles. The microbubbles formed by the fusion nanobubbles become echogenic once inside the tumor. Created with Biorender; Adapted from [138], MDPI, 2020.

The Department of Bioengineering at the University of Utah has developed an echogenic drug delivery system. The process is composed of two types of nanoparticles, polymeric micelles and perfluoropentane nanobubbles, which contain doxorubicin. This lipophilic drug is located inside the micelles body and the nanobubbles wall. These micelles accumulate in tumors interstices via the EPR effect. Once integrated into the tumor tissues, the small nanobubbles merge into larger, highly echogenic microbubbles that provide a strong and long-lasting tumor contrasted by ultrasonography (Figure 6). Ultrasound treatment (sonication for 150 s at 3 megahertz MHZ) causes a significant increase in the number of drugs from micelles and micro/nanobubbles ingested by tumor cells, notably by disrupting cell membranes and promoting the diffusion of nanoparticles and nanoparticles-free drugs in tumor tissues. This method has been tested in mice bearing tumor xenografts of breast cancer and ovarian carcinoma [128,138]

Interestingly, Fuente et al. have reported a new technique for radiolabeling various N-(2-Hydroxypropyl) methacrylamide-Lauryl Methacrylate (HPMA-LMA-based micellar aggregates with hydrophobic oxine-complexes of the trivalent radiometals ^68^Ga and ^111^In. In vivo biodistibution in healthy organ mice results in slow polymeric micelles rupture in contact with blood serum, whereas their stability has been validated in a saline buffer. Since the hydrophobicity of radionuclide complexes was comparable to the hydrophobic drug, they have highlighted that polymeric micelle properties drive the strategies on drug transport [137]. Yang et al. also have described the low stability of the micelle particularly those from self-assembling once they are injected in the blood stream. To figure out this issue, they have described the preparation of unimolecular micelles (one micelle—one macromolecule) from polyamidoamine (PAMAM) dendrimers loaded with chemotherapeutics agent (e.g., doxorubicin) covalently bound through a hydrazone bond as a tumor selective theranostic platform. Due to their unimolecular design, the micelles described were more stable and were able to respond to pH-stimulus for drug release. After radiolabelling with ^64^Cu and conjugation with tumor-targeting peptide sequence, micelles were monitored by PET imaging in order to confirm their active targeting. The results obtained emphasized the use of this type of micelle for theranostic purposes [135].

#### 3.2.3. Micelles in Chemogene Co-Delivery for CRPC Therapy

It has been shown that micelles represent a candidate vehicle for both carriage and solubilisation of antitumor agents. In preclinical models of prostate cancer, the combination of docetaxel (antimitotic agent), rapamycin (mTOR inhibitor) and 17-N-allylamino-17-demethoxygeldanamycin (HSP90 inhibitor) in a single micellar system resulted in a more efficient inhibition of tumor growth in vitro compared to their individual efficacy and cytotoxic effects of the drugs were more effective with micellar-dependent delivery [139]

**Figure 7:**
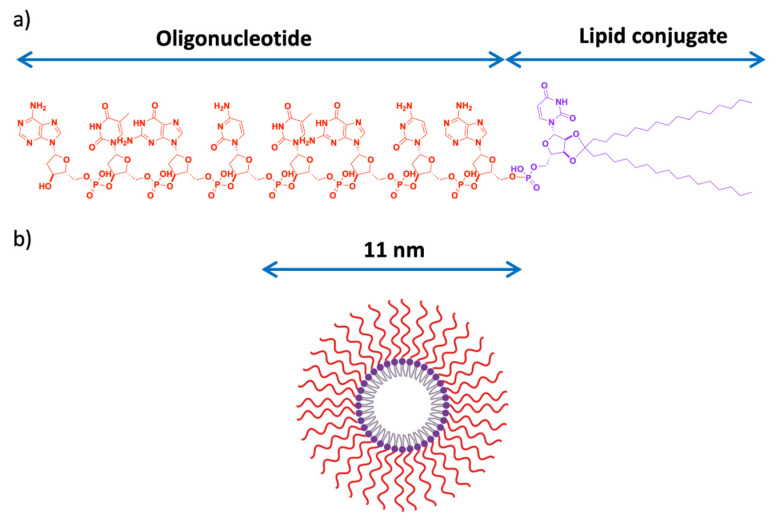
(**a**) Lipid-conjugated Antisense Oligonucleotide (LASO) structure; (**b**) Auto assembly—micelle formation of LASO in aqueous media; with a particle size of 11 nm. Adapted from [141], Elsevier, 2017.

Similarly, our laboratory had previously shown that Translationally Controlled Tumor Protein (TCTP) is overexpressed in CRPC and plays antiapoptotic and cytoprotection roles in response to ADT [12,140]. Inhibition of TCTP using Antisense Oligonucleotide (ASO) correlated well with tumor sensitivity to cytotoxic drug [12]. However, poor intracellular delivery of ASO has remained a serious challenge to this innovative approach. To overcome this hurdle, we developed (first word-wide) a lipid-Oligonucleotide conjugate (LASO) that can self-assemble into small particles, organized into nanomicelles in an aqueous media (Figure 7) [141]. The micellar core offers great opportunity to encapsulate antitumor drugs for improved efficacy. We established a proof of concept that this Lipid-ASO nanohybrid (LASO nanomicelles) is able to improve cellular uptake efficiency of ASO in tumor cells, enhance gene inhibition in vitro and decreased tumor volume in vivo [141]. In addition to gene control, LASO nanomicelles posit to reposition the delivery of chemogene therapies, taking advantage of its amphiphilic property. It is hoped that this innovative nanoconstruct potentiates a trend towards synergizing gene inhibition and cytotoxicity in a combinational strategy against CRPC.

### 3.3. Dendrimer Nanoparticles

**Figure 8:**
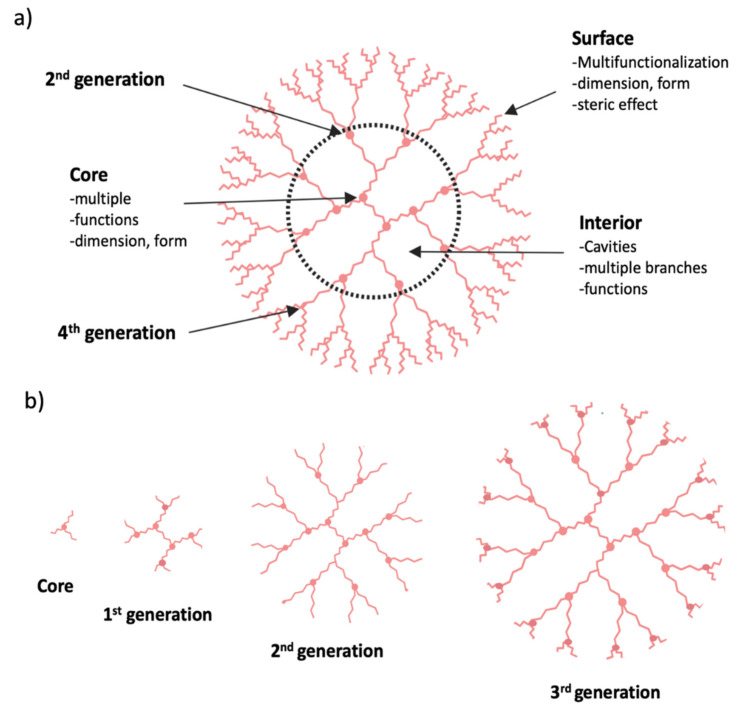
(**a**) Representation of the structure of a dendrimer; (**b**) Different stages of the divergent synthesis of a dendrimer created with Biorender; Adapted from [143], Drug Discovery Today, 2001.

Dendrimers are supramolecular polymeric vectors consisting of monomers that radially branch off from the central core. The construction is carried out by repeating the same reactions sequence until new identical branch generation is obtained at the end of each cycle. After a few generations, the dendrimer generally takes on a spherical, highly branched and multi-functional form, thanks to the numerous terminal functions present at the periphery (Figure 8a). Two types of syntheses are possible. Divergent synthesis occurs from the core to the periphery, adding more and more small molecules onto the surface of the dendrimer (Figure 8b). Convergent synthesis takes place from the periphery to the core, using dendritic fragments called Dendrons that are attached to a multifunctional core during a final step [142].

The dendrimers have a nanometric size and a symmetrical shape. They can be synthesized identically on a large scale. These unique features make dendrimers attractive for biomedical applications such as drug delivery. Thanks to this easy and controlled fabrication, different generations of dendrimer synthesis can be exploited to fulfill the desired pharmacokinetic requirements in vivo [144].

#### 3.3.1. Mechanism of Action of Dendrimers

**Figure 9:**
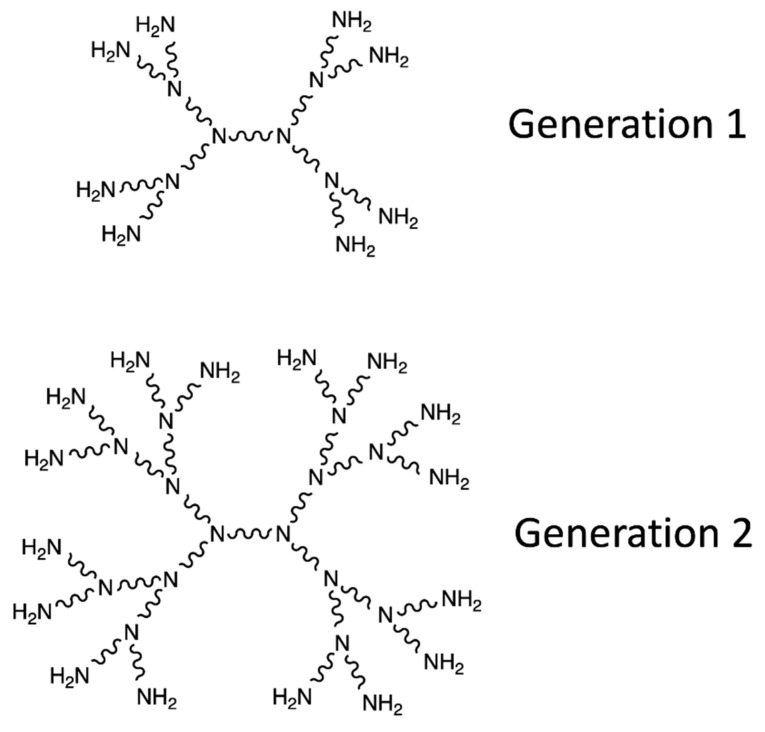
Tetrafunctional synthesis of polyamidoamine (PAMAM). Comprehensive Michael addition of amine groups with methyl acrylate, followed by amidation of the ester resulting from ethylenediamine. Adapted and modified from [143], Drug Discovery Today, 2001.

The rapid emergence of water soluble and biocompatible dendrimers came with an increase in their diversity. Polyamidoamine dendrimers (PAMAM), prepared by the divergent growth approach, are widely used in biology. Surface groups can be linked to targeting moieties, imaging probes, and therapeutic agents [142]. PAMAM dendrimers have primary and tertiary amine groups, which differ in their pKa values. Primary amine groups at the surface participate in DNA binding and cellular uptake of these complexes (Figure 9). However, indoor tertiary amine groups have a buffer effect in the endosome and improve the release of DNA into the cytoplasm. The nanocomposite Fe_3_O_4_/Au-Ac-AF, with folic acid (AF) mediated targeting [145] is an example of PAMAM dendrimers. It is able to be specifically endocytosed by cancer cells overexpressing AF receptors and to be used as a nanoprobe. The relatively hydrophobic core of the dendrimers can be used to effectively encapsulate hydrophobic anti-cancer drugs. It is possible, for example, to encapsulate anticancer drugs α-Tos (folic acid alpha-tocopherol succinate) [146].

PAMAM dendrimers are also able to regulate transportation across the epithelial barrier, indicating their potential for oral administration [147]. The modulation of the dendrimers makes it possible to optimize the adaptability of each function. This makes these nanostructures interesting for the passive and active vectorization of theranostic agents [148].

#### 3.3.2. Clinical Significance of Dendrimer

Dendrimer has been very useful for several clinical applications Its unique characteristics such as multivalency, variable chemical compositions as well as high biological compatibility make it suitable for drug delivery purposes and imaging. Their ability to confirm to diverse surface modifications and interaction with charged functional groups makes them excellent tool for drug discovery. Furthermore, dendrimers have emerged an excellent MRI contrast agent. In addition, their involvement in the design of electrochemical detectors is an attractive area of research rapidly evolving to lend support in the quick diagnosis of diseases [149].

## 4. Immunologic Response and Nanovectorization-Based Drug Delivery

Apart from the ability to deliver multiple drug agents at the same time, nanovectors constitute a formidable tool for immune targeting due to their preferential uptake by monocytes, macrophages, and dendritic cells within the body upon delivery. They can serve both as immunomodulation agents themselves and as a platform for delivery of immunomodulation agents. Different nanovectors can be designed to possess intrinsic immunomodulatory properties that can trigger antitumor immune response. Thus, whether through the design of more-efficient delivery devices for immunomodulation agents or the engineering of sophisticated nanoconstructs that can selectively regulate immune cell functions, immune nanomedicine represents an exciting opportunity to develop effective strategies that may one day significantly improve cancer treatment. For example, the surface decoration of nanoparticles can be further modified with antibodies, peptides, or recombinant proteins that further enhance the selective accumulation of drugs within tumor tissues. These unique advantages of nanomaterials have also been adopted for immuno-oncology applications [150].

## 5. Nanotheranostic Approach for CRPC Therapy

**Figure 10:**
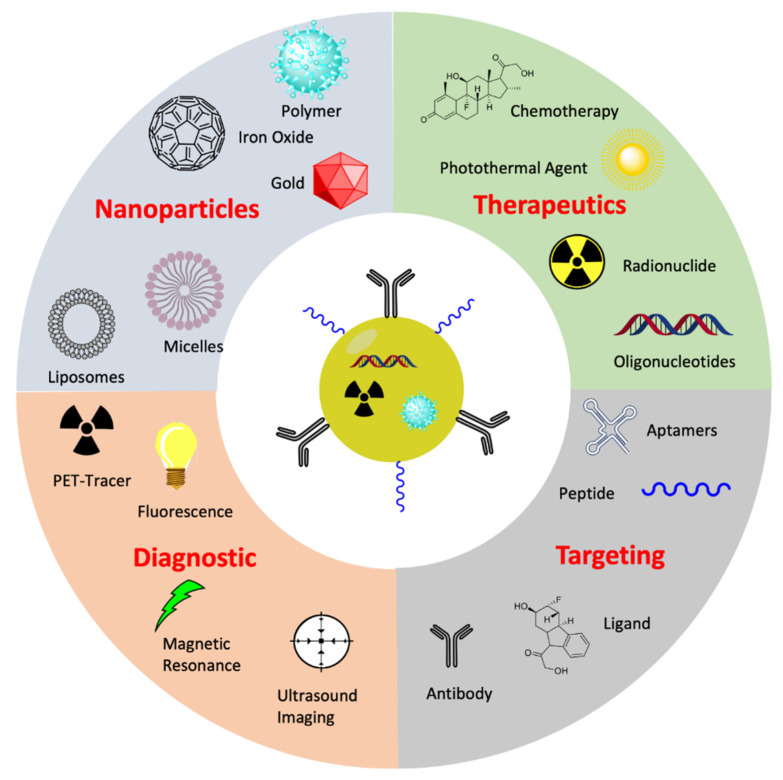
Schematic drawing of nanoparticles used for multimodal imaging and therapy (created with Biorender).

The term ‘theranostics’ corresponds to the combination of therapeutics (thera) and diagnostics (nostics) together for individualized disease management. Recent progress in the field of nanotechnologies has provided new multimodal nanotheranostics platforms bearing different properties (Figure 9). Theranostic nanoplatforms facilitates diagnostics, therapeutics, and real time monitoring of tissue response. They are designed to provide multiple features, including imaging, targeting and delivery characteristics for improving the therapeutic potential. Development of theranostic nanoplatforms requires several components with different properties [151]. Hence, several nanoparticles for radionuclide imaging [152,153,154,155,156,157,158] and therapy [159,160,161] featuring different functionalities have been investigated (Figure 10).

Theranostic nanoparticles are designed to improve therapeutic efficacy and to reduce side effects. In this context, an important aim relies on the tumor penetration and the improvement of therapeutics performance. In order to induce the accumulation of nanoparticles at the tumor site, the nanoparticles should be designed to take advantage of the permeability and retention effect (EPR) [162]. Here, the nanoparticles are used to improve the efficacy of chemotherapy and reduce side toxic effects. Different strategies have emerged for improving tumor penetration such as specific targeting, intratumoral delivery or regulating the tumor microenvironement and vasculature. Together, these achievements lead to better tumor penetration features and improve therapeutic efficiency [163].

## 6. Studies on Nanovectorization of Chemical Drugs for CRPC Treatment

**Figure 11:**
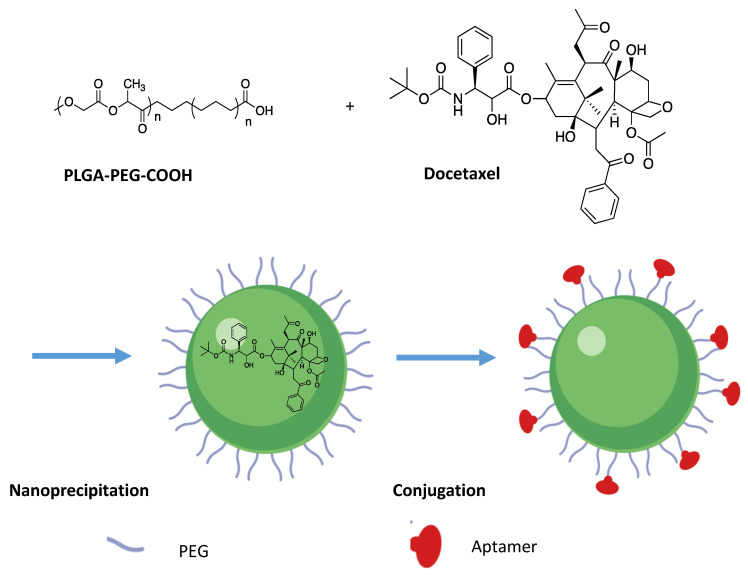
Synthesis of the nanoparticle PLGA-b-PEG-COOH: Docetaxel is encapsulated using the nanoprecipitation method. The nanoparticle is then covalently conjugated to aptamer (Apt) A10. Created with Biorender; modified from [167], Springer Science & Business Media; 2013.

The first approaches to nanovectorization against prostate cancer have been developed for chemotherapeutic agents already used for CRPC. This is particularly the case for docetaxel (see above), for which several nanovectors are under study. A nanovectorization strategy for this drug is the use of nanoparticles composed of PLGA-PEG polymers forming a micelle containing docetaxel (Figure 11). Polymers are conjugated to the A10 RNA aptamer, which targets a membrane antigen specifically expressed by tumor cells of prostate cancer, thereby significantly enhancing the drug’s contribution to the tumor tissue [164,165]. It has furthermore been demonstrated that the concentration of polymers used for the synthesis of the nanoparticles is linearly dependent on their size obtained. Moreover, this size appears to be a determining factor for the bio-distribution and clinical development of targeted therapies [164]. Bind-therapeutics has developed this treatment under the name Bind-014. It is currently in phase 2 clinical trial and the first results are promising in terms of the effectiveness of treatment as well as tolerance and safety. A reduction in the side effects that usually limit the dose of docetaxel given conventionally has been observed [166]. A nanovectorization strategy for docetaxel trihydrate, DEPTM docetaxel, using dendrimers was carried out in phase 1 clinical trials by the company Starpharma [166].

**Figure 12:**
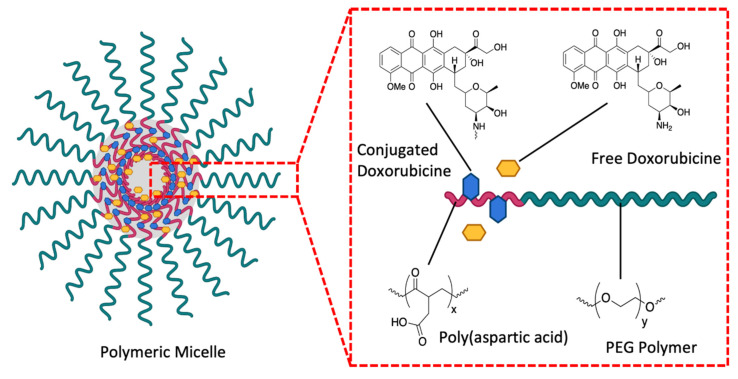
Schematic structure of NK911. A polymeric micelle composed of a copolymer block of PEG and polyaspartic acid (about 30 units). The polyethylene glycol is located at the outer level of the shell of the micelle. NK911 has a very hydrophobic heart, which allows it to capture a sufficient amount of doxorubicin, created with Biorender; adapted and modified from [170], Br J Cancer, 2004.

Furthermore, nanovectorization has also allowed for the use of doxorubicin, a chemotherapeutic agent that previously did not appear to be suitable for prostate cancer. Doxorubicin is indicated for the treatment of leukemias, lymphomas and bone sarcomas. It damages the DNA of the targeted cells with the intercalation of its anthracycline part, the chelation of metal ions or the generation of free radicals [168]. However, when used against prostate cancer, the drug is rapidly eliminated from the blood stream and too little tumor accumulation is observed. Classically administered, the drug also has a fairly high toxicity that seems to be due to its large volume of distribution with adverse side effects, including cardiotoxicity and myelosuppression [169]. Research on the nanovectorization of this drug follows the rise in prostate cancer resistant to current hormonal treatments and the demand for new effective molecules. The NK911 nanovectorization system was carried to phase 1 of clinical trial [170]. As described above, it is a micelle composed of copolymers of PEG and polyaspartic acid (Figure 12). This system of nanovectorization lowers the toxicity of the drug; however, its effectiveness remains to be improved. One possibility would be to use it as part of chemotherapy in combination with other agents [111].

## 7. Studies on Nanovectorization of Therapeutic Oligonucleotides for CRPC Treatment

The emergence of gene therapy treatments such as RNA interference (RNAi) or Antisense Oligonucleotides (ASO) raises many hopes. However, current studies show that these technologies are still limited by the low permeability of the cells to nucleic acids and the low stability of the latter with respect to serum proteins and degradation enzymes, especially for small interfering RNAs (siRNA for “small-interfering RNA”) [171]. One solution to these problems is the use of efficient vector systems that protect nucleic acids from nucleases present in body fluids and increase the permeability of the plasma membrane to these therapeutic agents. Viral vectors have initially emerged as an effective means of vectorizing nucleic acids; however, their potential inflammatory, immunogenic and mutagenic effects make them less effective and stress the urgent need for alternative non-viral vectors. Cationic polymers and lipids are the two most common nonviral vectors for gene therapy. They have the ability to form stable complexes with them via electrostatic interactions [172].

Chitosan is a cationic polymer that exhibits low cytotoxicity, strong biocompatibility and for which cells are highly permeable. It is frequently used for the administration of therapeutic nucleic acid. Nevertheless, present-day actual chitosan-based vectors have in vivo toxicity and a low release efficiency of nucleic acids. In order to overcome these shortcomings, research is currently underway on the development of a nanocomplex of this polymer in its natural form, conjugated with protamine, lecithin, and thiamine pyrophosphate for the vectorization of siRNA targeting the Survivin (SVN) gene [171]. This gene codes for an inhibitor of apoptosis (SVN), an attractive target for the treatment of prostate cancer. In vitro, this vector (GP-L-CT) reduces SVN expression by up to 22% in human prostate cancer cells. The GP-L-CT tumor growth and targeting inhibition efficiency also observed in vivo in mice carrying a PC-3 xenograft makes this vector a good alternative to other formulations composed of polymer nanoparticles. Its use as a therapeutic and theranostic system against prostate cancer is conceivable [171].

**Figure 13:**
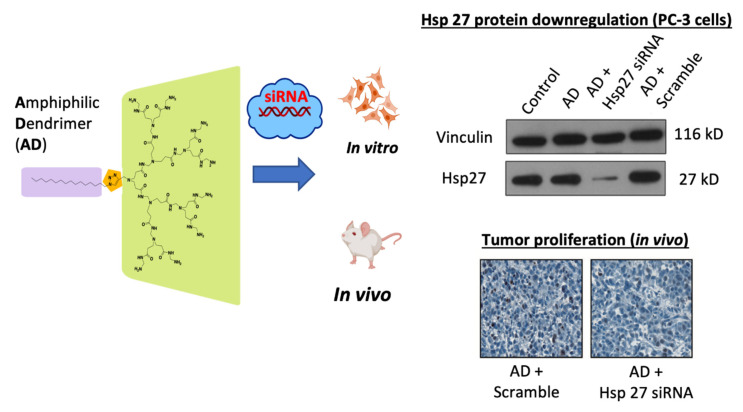
Structure and anti-cancer effect of the AmDM vector used with a siRNA targeting Hsp27. The AmDm vector is an amphiphilic dendrimer (in violet the hydrophobic alkyl chain, in green the dendrimer part of PANAM hydrophilic). Hsp27 protein is significantly under-translated and tumor proliferation reduced with treatment [173], Pharm Res. 2014.

Lipid vectors are presumed to deliver nucleic acid release through a membrane fusion mechanism, while polymeric vectors utilize the proton sponge effect to escape from the endosome (in which the acid pH eventually denatures the endosome). Therapeutic nucleic acids ingested by the cell). An amphiphilic Dendron dendrimer of PANAM can combine the advantages of both types of vectors [172]. This dendrimer is a kind of lipid/dendrimer hybrid that consists of a long alkyl chain and a dendrimer part. It has been tested as a vector of a siRNA repressing the translation of Hsp27, a gene coding for a chaperone protein that plays an important role for CRPC progression. Inhibition of its translation induces apoptosis and inhibits cell proliferation in vitro [173] Administration of this treatment to mice significantly lowered the translation of Hsp27 and produced a potent anti-cancer effect (Figure 13). This system offers a new alternative to castration-resistant prostate cancer (CRPC) for which there is still no effective treatment.

Nanovectors of nucleic acids that can be used against prostate cancer are at a fairly advanced stage of development. This is particularly the case of the SGT-53 system developed by SynerGeneTherapeutics, in which the tumor suppressor gene p53 is vectorized within a liposome. This treatment is currently in Phase 1 clinical trial. The TCTP-LASO system intended for use against CRPCs is based on the self-assembly of micellar nanoparticles of antisense nucleotides coupled to a lipid chain. The antisense oligonucleotide of this treatment targets the TCTP protein (translationally controlled tumor protein) involved in the cytoprotection role of Hsp27. It was the subject of a filing of a patent by a consortium of several French laboratories [174].

## 8. Conclusions

Today, interplay between technology and biology has tremendously impacted drug design, drug delivery and disease control. The advent of nanotherapeutics in the last few decades has reinvigorated and reshaped cancer treatment such that delivery of antitumor agents can now be controlled using nanovectors, resulting in decreased adverse effects and enhanced efficacy. As a result, nanovectorization has emerged as an attractive strategy in cancer therapy.

While new nanovectors are undergoing developmental stages, micelles, liposomes and dendrimers have been widely studied and utilized a great deal for treatment of various disease conditions including cancer. Even though research has progressed very rapidly, lending the concepts of nanotechnology to the treatment of many cancer types, only a few preclinical studies on nanovectorization-based therapy has focused on prostate tumors.

This review has tried to bring abreast some of the nanovectors that are currently being used as drug delivery systems in cancer treatment with specific possibilities on how they can be specifically utilized to transform prostate cancer therapy. The review started with the description and classification of the nanovectors, continued with how these vectors are adapted to their various mechanisms of delivery of antitumor agents with highlights on their structural characteristics that may render them excellent drug carriers for prostate cancer treatment as well, using gene delivery strategies, especially for the refractory subtypes. It then bottled up by drawing both clinical and preclinical proofs demonstrating the benefits and prospects of nanovectorization in the disease context such as prostate cancer. Whereas the majority of the first generation nanovectors have shown substantial clinical benefits through the passive targeting mechanism of cancer drugs delivery, nanovectors based on active targeting strategy are still hurdling to the clinic.

Given the era where genotherapy is taking a center stage in cancer treatment, either in combination with chemotherapeutic agent or as a monotherapy and has shown strong promise to favor CRPC through targeting genes that are activated by androgen withdrawal, poor cellular uptake, non-specificity and systemic instability have been ostensibly intractable. Nanovectorization proffers a lasting solution to the triads of challenge associated with, not only prostate cancer genotherapy, but also with other cancer types.

We are, indeed, hopeful that, (1) with the high level of interest and speed of research growing in different laboratories on nanovectorization, (2) with the promises of nanovectorization in cancer therapy, (3) with a number of pharmaceutical companies showing strong interest to hack into nanovectorization strategies for different pharmacological applications, nanotherapeutics is set to shift the paradigm of cancer therapy from traditional delivery to tumor-specific, active targeting delivery and concomitant real time in vivo imaging that will revolutionize both oncological and non-oncological therapies.

## Figures and Tables

**Table 2:** Selected approved and experimental therapeutics agents for CRPC targeting non-AR axis (clinicaltrials.gov, accessed on 10 February 2021), adapted from Reference [1].

	Stress Response Pathways	Signals Transduction Targets	Cellular Proliferative Targets	Tumor Microenvironment
Targets	Clusterin, HSP90, Bcl-2, HSP27	PI3K, Akt, mTOR, eIF4E, IGF-IR	Microtubules, PARP1, SERCA Pump	Neurotransmitters, somatostatin, endoglins, VEGF/FGFR, α-integrin
Approved therapy	-	-	Docetaxel, cabaxitacel	Denosumab, Radium-223
Experimental therapy	OGX-011, OGX-427	BEZ235, BKM120, AZD6363, MK2206, AZD8186, Linsitinib, Lapatinib	Tesetaxel, Patupilone, Ixabepilone, G-202	Sibrotuzumab, TRC-105, EMD525797, BMTP-11, Dovitinib, Beracizumab, pazopanib, phenelzine, pasireotide

Abbreviations: PI3K, phosphatidylinositol triphosphate kinase; VEGF, vascular endothelial growth factor; mTOR, mammalian target of rapamycin; PARP, poly-ADP ribose polymerase; SERCA, sarcoplasmic/endoplasmic reticulum calcium adenosine triphosphatase.

**Table 3:** Tabulation of the Nanoparticle currently used for prostate cancer, their bioactive ingredients and targets.

Generation	Particle	Targeting	Loading	Ref
First	Liposome ^1^	EPR	Celecoxib/Genistein	[83]
	Polymeric ^2^/Nanobuble	EPR	Curcumin	[84]
	Liposome	EPR	PEG (avoid RES ^3^ uptake)	[59]
Second	Liposome ^4^	Apatamer	TFO ^5^	[85]
	Liposome ^6^	Peptide	Doxorubicine/Vinorebline	[86]
	Liposome ^7^	Antibody ^8^	Doxorubicine	[87]
Third	DNA nanostructure	Apatamer	Doxorubicine	[88]
	PMB nanoparticle ^9^	Small Molecule ^10^	Reservatrol/Docetaxel	[89]
	Liposome	RGD	siRNA ^11^/Docetaxel	[90]
	Gold Nanoparticle	Small Molecule ^10^	siRNA	[91]

^1^ eggPC:L-α-phosphatidylcholine/DPPE-PEG-2000: 1,2-dipalmitoyl-sn-glycero-3- phosphoethanolamine-N- [methoxy(polyethylene glycol)-2000] ammonium salt; ^2^ Dextrane; ^3^ Reticuloendothelial system; ^4^ PLGA: poly(lactic-co-glycolic acid); ^5^ triple forming oligonucleotide; ^6^ PEGylated Lipid; ^7^ Soybean phosphatidylcholine (SPC), cholesterol, and DSPE-PEG_2000_-NHS; ^8^ simvastatin; ^9^ Planetary ball milled nanoparticles; ^10^ Folic Acid; ^11^ GRP 78 siRNA.

**Table 4:** Tabulation of the nanovectorization-based drugs in clinical trials for prostate cancer treatment, adapted from Reference [92].

Drug	Nanoformulation	Phase	Trial Status	Clinicaltrial.Gov Identifier
Curcumin	Nanomicellar gel	Second phase	ongoing	NCT02724618
Paclitaxel Lapatinib	Albumin NP	First	Completed	NCT00313599
siRNA for inhibition of M2 subunit of Ribonucleotide reductase (R2)	Cyclodexrin containing polymer stabilized by PEG	First	Terminated in 2013	NCT00689065
2-Hydroxyl Flutamide (2-HOF)	Calcium sulphate gel	second	completed	NCT02341404
M-VM3 (TLR5-receptor and its agonist protein 502s)	Adenoviral	First	Ongoing	NCT02654938
IL-12	Adenoviral	First	completed	NCT00406939

## References

[1] Toren P.J., Gleave M.E. (2013). Novel non-AR therapeutic targets in castrate resistant prostate cancer. Transl. Androl. Urol..

[2] Armstrong A.J. (2018). Updates in advanced prostate cancer 2018. Prostate Cancer Prostatic Dis..

[3] Blankfield R.P. (2012). Androgen deprivation therapy for prostate cancer and cardiovascular death. JAMA.

[4] Lee C.-H., Kantoff P. (2019). Treatment of Metastatic Prostate Cancer in 2018. JAMA Oncol..

[5] Karantanos T., Corn P.G., Thompson T.C. (2013). Prostate cancer progression after androgen deprivation therapy: Mechanisms of castrate resistance and novel therapeutic approaches. Oncogene.

[6] Wadosky K.M., Koochekpour S. (2016). Molecular mechanisms underlying resistance to androgen deprivation therapy in prostate cancer. Oncotarget.

[7] Nadiminty N., Gao A.C. (2012). Mechanisms of persistent activation of the androgen receptor in CRPC: Recent advances and future perspectives. World J. Urol..

[8] Yuan X., Balk S.P. (2009). Mechanisms mediating androgen receptor reactivation after castration. Urol. Oncol. Semin. Orig. Investig..

[9] Dhavale M., Abdelaal M.K., Alam A.B.M.N., Blazin T., Mohammed L.M., Prajapati D., Ballestas N.P., Mostafa J.A. (2021). Androgen Receptor Signaling and the Emergence of Lethal Neuroendocrine Prostate Cancer With the Treatment-Induced Suppression of the Androgen Receptor: A Literature Review. Cureus.

[10] Katsogiannou M., Ziouziou H., Karaki S., Andrieu C., de Villeneuve M., Rocchi P. (2015). The hallmarks of castration-resistant prostate cancers. Cancer Treat. Rev..

[11] Rocchi P., Beraldi E., Ettinger S., Fazli L., Vessella R.L., Nelson C., Gleave M. (2005). Increased Hsp27 after androgen ablation facilitates androgen-independent progression in prostate cancer via signal transducers and activators of transcription 3-mediated suppression of apoptosis. Cancer Res..

[12] Baylot V., Katsogiannou M., Andrieu C., Taieb D., Acunzo J., Giusiano S., Fazli L., Gleave M., Garrido C., Rocchi P. (2012). Targeting TCTP as a new therapeutic strategy in castration-resistant prostate cancer. Mol. Ther. J. Am. Soc. Gene Ther..

[13] Colombel M., Symmans F., Gil S., O’Toole K.M., Chopin D., Benson M., Olsson C.A., Korsmeyer S., Buttyan R. (1993). Detection of the apoptosis-suppressing oncoprotein bc1-2 in hormone-refractory human prostate cancers. Am. J. Pathol..

[14] Hollenhorst P.C., Paul L., Ferris M.W., Graves B.J. (2011). The ETS gene ETV4 is required for anchorage-independent growth and a cell proliferation gene expression program in PC3 prostate cells. Genes Cancer.

[15] Suzuki H., Freije D., Nusskern D.R., Okami K., Cairns P., Sidransky D., Isaacs W.B., Bova G.S. (1998). Interfocal heterogeneity of PTEN/MMAC1 gene alterations in multiple metastatic prostate cancer tissues. Cancer Res..

[16] Sette C. (2013). Alternative Splicing Programs in Prostate Cancer. Int. J. Cell Biol..

[17] Chin S.P., Dickinson J.L., Holloway A.F. (2011). Epigenetic regulation of prostate cancer. Clin. Epigenet..

[18] Nelson M., Dornbier R., Kirshenbaum E., Eguia E., Sweigert P., Baker M., Farooq A., McVary K.T., Gonzalez C.M., Gupta G. (2019). Utilization of Surgery for Post-prostatectomy Incontinence. J. Urol..

[19] Herrera F.G., Berthold D.R. (2016). Radiation Therapy after Radical Prostatectomy: Implications for Clinicians. Front. Oncol..

[20] Brawer M.K. (2006). Hormonal Therapy for Prostate Cancer. Rev. Urol..

[21] Nader R., El Amm J., Aragon-Ching J.B. (2018). Role of chemotherapy in prostate cancer. Asian J. Androl..

[22] Lin M.-C., Wang M., Chou M.-C., Chao C.-N., Fang C.-Y., Chen P.-L., Chang D., Shen C.-H. (2019). Gene therapy for castration-resistant prostate cancer cells using JC polyomavirus-like particles packaged with a PSA promoter driven-suicide gene. Cancer Gene Ther..

[23] Galsky M.D., Vogelzang N.J. (2010). Docetaxel-based combination therapy for castration-resistant prostate cancer. Ann. Oncol..

[24] Sriraman S.K., Aryasomayajula B., Torchilin V.P. (2014). Barriers to drug delivery in solid tumors. Tissue Barriers.

[25] Zhou M., Li L., Li L., Lin X., Wang F., Li Q., Huang Y. (2019). Overcoming chemotherapy resistance via simultaneous drug-efflux circumvention and mitochondrial targeting. Acta Pharm. Sin. B.

[26] Gillet J.-P., Gottesman M.M. (2010). Mechanisms of multidrug resistance in cancer. Methods Mol. Biol..

[27] Narvekar M., Xue H.Y., Eoh J.Y., Wong H.L. (2014). Nanocarrier for poorly water-soluble anticancer drugs–barriers of translation and solutions. AAPS PharmSciTech.

[28] Hatefi A., Amsden B. (2002). Camptothecin Delivery Methods. Pharm. Res..

[29] Ma P., Mumper R.J. (2013). Paclitaxel Nano-Delivery Systems: A Comprehensive Review. J. Nanomed. Nanotechnol..

[30] Price N., Jain V.K., Sartor O. (2004). Docetaxel Improves Survival in Metastatic Androgen-Independent Prostate Cancer. Clin. Prostate Cancer.

[31] Shelley M., Mason M.D. (2004). Docetaxel plus prednisone or mitoxantrone plus prednisone for advanced prostate cancer. N. Engl. J. Med..

[32] Andreopoulou E., Sparano J.A. (2013). Chemotherapy in Patients with Anthracycline- and Taxane-Pretreated Metastatic Breast Cancer: An Overview. Curr. Breast Cancer Rep..

[33] Zheng R., Han S., Duan C., Chen K., You Z., Jia J., Lin S., Liang L., Liu A., Long H. (2015). Role of Taxane and Anthracycline Combination Regimens in the Management of Advanced Breast Cancer. Medicine.

[34] Elm’hadi C., Tanz R., Khmamouche M.R., Toreis M., Mahfoud T., Slimani K.A., Errihani H., Ichou M. (2016). Toxicities of docetaxel: Original drug versus generics—a comparative study about 81 cases. Springerplus.

[35] Panday V.R.N., Huizing M.T., Ten Bokkel Huinink W.W., Vermorken J.B., Beijnen J.H. (1997). Hypersensitivity Reactions to the Taxanes Paclitaxel and Docetaxel. Clin. Drug Investig..

[36] Min B.-D., Kang H.-W., Kim W.-T., Kim Y.-J., Yun S.J., Lee S.C., Kim W.-J. (2012). Docetaxel-Induced Fatal Interstitial Pneumonitis in a Patient with Castration-Resistant Prostate Cancer. Korean J. Urol..

[37] Data Sheet 2005. https://web.archive.org/web/20051228000129/http://medsafe.govt.nz/Profs/datasheet/t/taxotereinf.htm.

[38] Webster T.J. (2006). Nanomedicine: What’s in a definition?. Int. J. Nanomed..

[39] Sakamoto J.H., van de Ven A.L., Godin B., Blanco E., Serda R.E., Grattoni A., Ziemys A., Bouamrani A., Hu T., Ranganathan S.I. (2010). Enabling individualized therapy through nanotechnology. Pharmacol. Res..

[40] Miao L., Guo S., Zhang J., Kim W.Y., Huang L. (2014). Nanoparticles with Precise Ratiometric Co-Loading and Co-Delivery of Gemcitabine Monophosphate and Cisplatin for Treatment of Bladder Cancer. Adv. Funct. Mater..

[41] Mou Q., Ma Y., Ding F., Gao X., Yan D., Zhu X., Zhang C. (2019). Two-in-One Chemogene Assembled from Drug-Integrated Antisense Oligonucleotides To Reverse Chemoresistance. J. Am. Chem. Soc..

[42] Liu S., Guo Y., Huang R., Li J., Huang S., Kuang Y., Han L., Jiang C. (2012). Gene and doxorubicin co-delivery system for targeting therapy of glioma. Biomaterials.

[43] Liu X., Liu C., Chen C., Bentobji M., Cheillan F.A., Piana J.T., Qu F., Rocchi P., Peng L. (2014). Targeted delivery of Dicer-substrate siRNAs using a dual targeting peptide decorated dendrimer delivery system. Nanomed. Nanotechnol. Biol. Med..

[44] Senapati S., Mahanta A.K., Kumar S., Maiti P. (2018). Controlled drug delivery vehicles for cancer treatment and their performance. Signal Transduct. Target. Ther..

[45] Zhang Y., Huang Y., Li S. (2014). Polymeric Micelles: Nanocarriers for Cancer-Targeted Drug Delivery. AAPS PharmSciTech.

[46] Raj K.K., Anil K.S., Rajesh K.K. (2016). Novel Approaches for Drug Delivery.

[47] Xiang Y., Bernards N., Hoang B., Zheng J., Matsuura N. (2019). Perfluorocarbon nanodroplets can reoxygenate hypoxic tumors in vivo without carbogen breathing. Nanotheranostics.

[48] Asem H., Malmström E. (2018). Polymeric Nanoparticles Explored for Drug-Delivery Applications. Gels and Other Soft Amorphous Solids American Chemical Society.

[49] Kröger A.P.P., Hamelmann N.M., Juan A., Lindhoud S., Paulusse J.M.J. (2018). Biocompatible Single-Chain Polymer Nanoparticles for Drug Delivery—A Dual Approach. ACS Appl. Mater. Interfaces.

[50] Inorganic Nanoparticles for Targeted Drug Delivery—ScienceDirect. https://www.sciencedirect.com/science/article/pii/B9781845695095500082.

[51] Guo Q., Shen X., Li Y., Xu S. (2017). Carbon nanotubes-based drug delivery to cancer and brain. Curr. Med. Sci..

[52] Villemin E., Ong Y.C., Thomas C.M., Gasser G. (2019). Polymer encapsulation of ruthenium complexes for biological and medicinal applications. Nat. Rev. Chem..

[53] Parveen S., Sahoo S.K. (2008). Polymeric nanoparticles for cancer therapy. J. Drug Target..

[54] Zamboni W.C. (2005). Liposomal, nanoparticle, and conjugated formulations of anticancer agents. Clin. Cancer Res. An Off. J. Am. Assoc. Cancer Res..

[55] Malam Y., Loizidou M., Seifalian A.M. (2009). Liposomes and nanoparticles: Nanosized vehicles for drug delivery in cancer. Trends Pharmacol. Sci..

[56] Uthaman S., Huh K.M., Park I.-K. (2018). Tumor microenvironment-responsive nanoparticles for cancer theragnostic applications. Biomater. Res..

[57] Maeda H., Wu J., Sawa T., Matsumura Y., Hori K. (2000). Tumor vascular permeability and the EPR effect in macromolecular therapeutics: A review. J. Control. Release Off. J. Control. Release Soc..

[58] Akhtar M.J., Ahamed M., Alhadlaq H.A., Alrokayan S.A., Kumar S. (2014). Targeted anticancer therapy: Overexpressed receptors and nanotechnology. Clin. Chim. Acta..

[59] Milla P., Dosio F., Cattel L. (2012). PEGylation of proteins and liposomes: A powerful and flexible strategy to improve the drug delivery. Curr. Drug Metab..

[60] Raj T.A.S., Smith A.M., Moore A.S. (2013). Vincristine sulfate liposomal injection for acute lymphoblastic leukemia. Int. J. Nanomedicine.

[61] Wang Y., Cao X., Guo R., Shen M., Zhang M., Zhu M., Shi X. (2011). Targeted delivery of doxorubicin into cancer cells using a folic acid–dendrimer conjugate. Polym. Chem..

[62] Barenholz Y. (2012). Doxil^®^–the first FDA-approved nano-drug: Lessons learned. J. Control. Release Off. J. Control. Release Soc..

[63] Moghimi S.M., Simberg D. (2018). Nanoparticle transport pathways into tumors. J. Nanopart. Res..

[64] Fang J., Nakamura H., Maeda H. (2011). The EPR effect: Unique features of tumor blood vessels for drug delivery, factors involved, and limitations and augmentation of the effect. Adv. Drug Deliv. Rev..

[65] Matsumura Y., Maeda H. (1986). A New Concept for Macromolecular Therapeutics in Cancer Chemotherapy: Mechanism of Tumoritropic Accumulation of Proteins and the Antitumor Agent Smancs. Cancer Res..

[66] Maeda H. (2012). Macromolecular therapeutics in cancer treatment: The EPR effect and beyond. J. Control. Release.

[67] Blanco E., Shen H., Ferrari M. (2015). Principles of nanoparticle design for overcoming biological barriers to drug delivery. Nat. Biotechnol..

[68] Couvreur P. (2013). Les nanotechnologies peuvent-elles contribuer à traiter des maladies sévères?: Chaire d’Innovation Technologique Liliane Bettencourt 2009-2010. Leçon prononcée le jeudi 21 janvier 2010. Les Nanotechnologies Peuvent-Elles Contribuer à Traiter des Maladies Sévères?.

[69] Yu B., Tai H.C., Xue W., Lee L.J., Lee R.J. (2010). Receptor-targeted nanocarriers for therapeutic delivery to cancer. Mol. Membr. Biol..

[70] Baron J., Wang E.S. (2018). Gemtuzumab ozogamicin for the treatment of acute myeloid leukemia. Expert Rev. Clin. Pharmacol..

[71] Bareford L.M., Swaan P.W. (2007). Endocytic mechanisms for targeted drug delivery. Adv. Drug Deliv. Rev..

[72] Xu S., Olenyuk B.Z., Okamoto C.T., Hamm-Alvarez S.F. (2013). Targeting receptor-mediated endocytotic pathways with nanoparticles: Rationale and advances. Adv. Drug Deliv. Rev..

[73] Katsogiannou M., Peng L., Catapano C.V., Rocchi P. (2011). Active-targeted nanotherapy strategies for prostate cancer. Curr. Cancer Drug Targets.

[74] Nahta R. (2012). Molecular Mechanisms of Trastuzumab-Based Treatment in HER2-Overexpressing Breast Cancer. ISRN Oncol..

[75] Marelli U.K., Rechenmacher F., Sobahi T.R.A., Mas-Moruno C., Kessler H. (2013). Tumor Targeting via Integrin Ligands. Front. Oncol..

[76] Yang Y., Zhang Y., Cao Z., Ji H., Yang X., Iwamoto H., Wahlberg E., Länne T., Sun B., Cao Y. (2013). Anti-VEGF– and anti-VEGF receptor–induced vascular alteration in mouse healthy tissues. Proc. Natl. Acad. Sci. USA.

[77] Duda D.G., Batchelor T.T., Willett C.G., Jain R.K. (2007). VEGF-targeted cancer therapy strategies: Current progress, hurdles and future prospects. Trends Mol. Med..

[78] Haberkorn U., Eder M., Kopka K., Babich J.W., Eisenhut M. (2016). New Strategies in Prostate Cancer: Prostate-Specific Membrane Antigen (PSMA) Ligands for Diagnosis and Therapy. Clin. Cancer Res..

[79] Wüstemann T., Haberkorn U., Babich J., Mier W. (2019). Targeting prostate cancer: Prostate-specific membrane antigen based diagnosis and therapy. Med. Res. Rev..

[80] Felber M., Alberto R. (2015). 99mTc radiolabelling of Fe_3_O_4_–Au core–shell and Au–Fe_3_O_4_ dumbbell-like nanoparticles. Nanoscale.

[81] Felber M., Bauwens M., Mateos J.M., Imstepf S., Mottaghy F.M., Alberto R. (2015). 99mTc Radiolabeling and Biological Evaluation of Nanoparticles Functionalized with a Versatile Coating Ligand. Chem. A Eur. J..

[82] Yari H., Nkepang G., Awasthi V. (2019). Surface Modification of Liposomes by a Lipopolymer Targeting Prostate Specific Membrane Antigen for Theranostic Delivery in Prostate Cancer. Materials.

[83] Tian J., Guo F., Chen Y., Li Y., Yu B., Li Y. (2019). Nanoliposomal formulation encapsulating celecoxib and genistein inhibiting COX-2 pathway and Glut-1 receptors to prevent prostate cancer cell proliferation. Cancer Lett..

[84] Bessone F., Argenziano M., Grillo G., Ferrara B., Pizzimenti S., Barrera G., Cravotto G., Guiot C., Stura I., Cavalli R. (2019). Low-dose curcuminoid-loaded in dextran nanobubbles can prevent metastatic spreading in prostate cancer cells. Nanotechnology.

[85] Jiao J., Zou Q., Zou M.H., Guo R.M., Zhu S., Zhang Y. (2016). Aptamer-modified PLGA nanoparticle delivery of triplex forming oligonucleotide for targeted prostate cancer therapy. Neoplasma.

[86] Yeh C.-Y., Hsiao J.-K., Wang Y.-P., Lan C.-H., Wu H.-C. (2016). Peptide-conjugated nanoparticles for targeted imaging and therapy of prostate cancer. Biomaterials.

[87] Li N., Xie X., Hu Y., He H., Fu X., Fang T., Li C. (2019). Herceptin-conjugated liposomes co-loaded with doxorubicin and simvastatin in targeted prostate cancer therapy. Am. J. Transl. Res..

[88] Taghdisi S.M., Danesh N.M., Ramezani M., Yazdian-Robati R., Abnous K. (2018). A Novel AS1411 Aptamer-Based Three-Way Junction Pocket DNA Nanostructure Loaded with Doxorubicin for Targeting Cancer Cells in Vitro and in Vivo. Mol. Pharm..

[89] Singh S.K., Lillard J.W., Singh R. (2018). Reversal of drug resistance by planetary ball milled (PBM) nanoparticle loaded with resveratrol and docetaxel in prostate cancer. Cancer Lett..

[90] Zhang X., He Z., Xiang L., Li L., Zhang H., Lin F., Cao H. (2019). Codelivery of GRP78 siRNA and docetaxel via RGD-PEG-DSPE/DOPA/CaP nanoparticles for the treatment of castration-resistant prostate cancer. Drug Des. Devel. Ther..

[91] Rahme K., Guo J., Holmes J.D., Dinesh Kumar L. (2019). Bioconjugated Gold Nanoparticles Enhance siRNA Delivery in Prostate Cancer Cells. RNA Interference and Cancer Therapy: Methods and Protocols.

[92] Gupta S., Gupta P.K., Dharanivasan G., Verma R.S. (2017). Current prospects and challenges of nanomedicine delivery in prostate cancer therapy. Nanomedicine.

[93] Abraham S.A., Waterhouse D.N., Mayer L.D., Cullis P.R., Madden T.D., Bally M.B. (2005). The liposomal formulation of doxorubicin. Methods Enzymol..

[94] Mukherjee A. (2017). A Review on Liposomes and Polymeric Nanoparticles as Drug Delivery Vehicles to the Brain. https://www.elynsgroup.com/journal/article/a-review-on-liposomes-and-polymeric-nanoparticles.

[95] Panahi Y., Farshbaf M., Mohammadhosseini M., Mirahadi M., Khalilov R., Saghfi S., Akbarzadeh A. (2017). Recent advances on liposomal nanoparticles: Synthesis, characterization and biomedical applications. Artif. Cells Nanomed. Biotechnol..

[96] Boehlke L., Winter J.N. (2006). Sphingomyelin/cholesterol liposomal vincristine: A new formulation for an old drug. Expert Opin. Biol. Ther..

[97] Udhrain A., Skubitz K.M., Northfelt D.W. (2007). Pegylated liposomal doxorubicin in the treatment of AIDS-related Kaposi’s sarcoma. Int. J. Nanomed..

[98] Pujade-Lauraine E., Wagner U., Aavall-Lundqvist E., Gebski V., Heywood M., Vasey P.A., Volgger B., Vergote I., Pignata S., Ferrero A. (2010). Pegylated liposomal doxorubicin and carboplatin compared with paclitaxel and carboplatin for patients with platinum-sensitive ovarian cancer in late relapse. J. Clin. Oncol..

[99] Perez A.T., Domenech G.H., Frankel C., Vogel C.L. (2002). Pegylated Liposomal Doxorubicin (Doxil^®^) for Metastatic Breast Cancer: The Cancer Research Network, Inc., Experience. Proceedings of the Cancer Investigation.

[100] O’Brien M.E.R., Wigler N., Inbar M., Rosso R., Grischke E., Santoro A., Catane R., Kieback D.G., Tomczak P., Ackland S.P. (2004). Reduced cardiotoxicity and comparable efficacy in a phase III trial of pegylated liposomal doxorubicin HCl (CAELYX^TM^/Doxil^®^) versus conventional doxorubicin for first-line treatment of metastatic breast cancer. Ann. Oncol..

[101] Ghaferi M., Asadollahzadeh M.J., Akbarzadeh A., Shahmabadi H.E., Alavi S.E. (2020). Enhanced efficacy of PEGylated liposomal cisplatin: In vitro and in vivo evaluation. Int. J. Mol. Sci..

[102] Maeda O., Kajiyama H., Shibata K., Nakamura S., Kikkawa F. (2020). Pegylated liposomal doxorubicin/oxaliplatin chemotherapy can overcome cisplatin resistance in spectrin αII-overexpressing ovarian carcinoma. Anticancer Res..

[103] Tannock I.F., de Wit R., Berry W.R., Horti J., Pluzanska A., Chi K.N., Oudard S., Théodore C., James N.D., Turesson I. (2004). Docetaxel plus prednisone or mitoxantrone plus prednisone for advanced prostate cancer. N. Engl. J. Med..

[104] Yaqub F. (2013). Mechanism of action of anthracycline drugs. Lancet Oncol..

[105] Narayanan N.K., Nargi D., Randolph C., Narayanan B.A. (2009). Liposome encapsulation of curcumin and resveratrol in combination reduces prostate cancer incidence in PTEN knockout mice. Int. J. Cancer.

[106] Thangapazham R.L., Puri A., Tele S., Blumenthal R., Maheshwari R.K. (2008). Evaluation of a nanotechnology-based carrier for delivery of curcumin in prostate cancer cells. Int. J. Oncol..

[107] Jantscheff P., Esser N., Graeser R., Ziroli V., Kluth J., Unger C., Massing U. (2009). Liposomal gemcitabine (GemLip)-efficient drug against hormone-refractory Du145 and PC-3 prostate cancer xenografts. Prostate.

[108] Bode C., Trojan L., Weiss C., Kraenzlin B., Michaelis U., Teifel M., Alken P., Michel M.S. (2009). Paclitaxel encapsulated in cationic liposomes: A new option for neovascular targeting for the treatment of prostate cancer. Oncol. Rep..

[109] Pinto A.C., Moreira J.N., Simões S. (2011). Liposomal imatinib–mitoxantrone combination: Formulation development and therapeutic evaluation in an animal model of prostate cancer. Prostate.

[110] Banerjee R., Tyagi P., Li S., Huang L. (2004). Anisamide-targeted stealth liposomes: A potent carrier for targeting doxorubicin to human prostate cancer cells. Int. J. Cancer.

[111] Harris K.A., Harney E., Small E.J. (2002). Liposomal doxorubicin for the treatment of hormone-refractory prostate cancer. Clin. Prostate Cancer.

[112] Fan X., Wang L., Guo Y., Xiong X., Zhu L., Fang K. (2016). Inhibition of prostate cancer growth using doxorubicin assisted by ultrasound-targeted nanobubble destruction. Int. J. Nanomed..

[113] Chen X., Wang X., Wang Y., Yang L., Hu J., Xiao W., Fu A., Cai L., Li X., Ye X. (2010). Improved tumor-targeting drug delivery and therapeutic efficacy by cationic liposome modified with truncated bFGF peptide. J. Control. Release Off. J. Control. Release Soc..

[114] Deeken J.F., Slack R., Weiss G.J., Ramanathan R.K., Pishvaian M.J., Hwang J., Lewandowski K., Subramaniam D., He A.R., Cotarla I. (2013). A phase I study of liposomal-encapsulated docetaxel (LE-DT) in patients with advanced solid tumor malignancies. Cancer Chemother. Pharmacol..

[115] Hagtvet E., Evjen T.J., Olsen D.R., Fossheim S.L., Nilssen E.A. (2011). Ultrasound enhanced antitumor activity of liposomal doxorubicin in mice. J. Drug Target..

[116] Hagtvet E., Røe K., Olsen D.R. (2011). Liposomal doxorubicin improves radiotherapy response in hypoxic prostate cancer xenografts. Radiat. Oncol..

[117] Jantscheff P., Ziroli V., Esser N., Graeser R., Kluth J., Sukolinskaya A., Taylor L.A., Unger C., Massing U. (2009). Anti-metastatic effects of liposomal gemcitabine in a human orthotopic LNCaP prostate cancer xenograft model. Clin. Exp. Metastasis.

[118] Pakunlu R.I., Wang Y., Saad M., Khandare J.J., Starovoytov V., Minko T. (2006). In vitro and in vivo intracellular liposomal delivery of antisense oligonucleotides and anticancer drug. J. Control. Release Off. J. Control. Release Soc..

[119] Şalva E., Turan S.Ö., Eren F., Akbuğa J. (2015). The enhancement of gene silencing efficiency with chitosan-coated liposome formulations of siRNAs targeting HIF-1α and VEGF. Int. J. Pharm..

[120] Bolotin E.M., Cohen R., Bar L.K., Emanuel N., Ninio S., Barenholz Y., Lasic D.D. (1994). Ammonium Sulfate Gradients for Efficient and Stable Remote Loading of Amphipathic Weak Bases into Liposomes and Ligandoliposomes. J. Liposome Res..

[121] Boman N.L., Masin D., Mayer L.D., Cullis P.R., Bally M.B. (1994). Liposomal vincristine which exhibits increased drug retention and increased circulation longevity cures mice bearing P388 tumors. Cancer Res..

[122] Structural Biochemistry/Lipids/Micelles—Wikibooks, Open Books for an Open World. https://en.wikibooks.org/wiki/Structural_Biochemistry/Lipids/Micelles.

[123] Blanco E., Kessinger C.W., Sumer B.D., Gao J. (2009). Multifunctional micellar nanomedicine for cancer therapy. Exp. Biol. Med..

[124] Nicolas J., Couvreur P. (2017). Polymer nanoparticles for the delivery of anticancer drug. Med. Sci. M/S.

[125] PEGylation in Anti-Cancer Therapy: An Overview—ScienceDirect. https://www.sciencedirect.com/science/article/pii/S1818087615000860.

[126] Suk J.S., Xu Q., Kim N., Hanes J., Ensign L.M. (2016). PEGylation as a strategy for improving nanoparticle-based drug and gene delivery. Adv. Drug Deliv. Rev..

[127] Thermosensitive and Biodegradable Polymeric Micelles for Paclitaxel Delivery. https://www.ncbi.nlm.nih.gov/pubmed/15763618.

[128] Nasongkla N., Bey E., Ren J., Ai H., Khemtong C., Guthi J.S., Chin S.-F., Sherry A.D., Boothman D.A., Gao J. (2006). Multifunctional polymeric micelles as cancer-targeted, MRI-ultrasensitive drug delivery systems. Nano Lett..

[129] Wiernik P.H., Schwartz E.L., Strauman J.J., Dutcher J.P., Lipton R.B., Paietta E. (1987). Phase I clinical and pharmacokinetic study of taxol. Cancer Res..

[130] Kim T.-Y., Kim D.-W., Chung J.-Y., Shin S.G., Kim S.-C., Heo D.S., Kim N.K., Bang Y.-J. (2004). Phase I and pharmacokinetic study of Genexol-PM, a cremophor-free, polymeric micelle-formulated paclitaxel, in patients with advanced malignancies. Clin. Cancer Res. An Off. J. Am. Assoc. Cancer Res..

[131] Alavi M., Hamidi M. (2019). Passive and active targeting in cancer therapy by liposomes and lipid nanoparticles. Drug Metab. Pers. Ther..

[132] Duncan R., Richardson S.C.W. (2012). Endocytosis and Intracellular Trafficking as Gateways for Nanomedicine Delivery: Opportunities and Challenges. Mol. Pharm..

[133] Mohanty C., Das M., Jagat R.K., Sanjeeb K.S. (2010). Receptor Mediated Tumor Targeting: An Emerging Approach for Cancer Therapy Current Drug Delivery. http://www.eurekaselect.com/72923/article.

[134] Neumann S.E., Chamberlayne C.F., Zare R.N. (2018). Electrically controlled drug release using pH-sensitive polymer films. Nanoscale.

[135] Yang J., Lu W., Xiao J., Zong Q., Xu H., Yin Y., Hong H., Xu W. (2018). A positron emission tomography image-guidable unimolecular micelle nanoplatform for cancer theranostic applications. Acta Biomater..

[136] Gulzar A., Gai S., Yang P., Li C., Ansari M.B., Lin J. (2015). Stimuli responsive drug delivery application of polymer and silica in biomedicine. J. Mater. Chem. B.

[137] de la Fuente A., Kramer S., Mohr N., Pektor S., Klasen B., Bausbacher N., Miederer M., Zentel R., Rösch F. (2019). 68Ga[Ga]-, 111In[In]-oxine: A novel strategy of in situ radiolabeling of HPMA-based micelles. Am. J. Nucl. Med. Mol. Imaging.

[138] (2007). Multifunctional Nanoparticles for Combining Ultrasonic Tumor Imaging and Targeted Chemotherapy News-Medical.net. https://www.news-medical.net/news/2007/08/22/28987.aspx.

[139] Le B., Powers G.L., Tam Y.T., Schumacher N., Malinowski R.L., Steinke L., Kwon G., Marker P.C. (2017). Multi-drug loaded micelles delivering chemotherapy and targeted therapies directed against HSP90 and the PI3K/AKT/mTOR pathway in prostate cancer. PLoS ONE.

[140] Baylot V., Karaki S., Rocchi P., Telerman A., Amson R. (2017). TCTP Has a Crucial Role in the Different Stages of Prostate Cancer Malignant Progression. TCTP/tpt1-Remodeling Signaling from Stem Cell to Disease.

[141] Karaki S., Benizri S., Mejías R., Baylot V., Branger N., Nguyen T., Vialet B., Oumzil K., Barthélémy P., Rocchi P. (2017). Lipid-oligonucleotide conjugates improve cellular uptake and efficiency of TCTP-antisense in castration-resistant prostate cancer. J. Control. Release Off. J. Control. Release Soc..

[142] Abbasi E., Aval S.F., Akbarzadeh A., Milani M., Nasrabadi H.T., Joo S.W., Hanifehpour Y., Nejati-Koshki K., Pashaei-Asl R. (2014). Dendrimers: Synthesis, applications, and properties. Nanoscale Res. Lett..

[143] Esfand R., Tomalia D.A. (2001). Poly(amidoamine) (PAMAM) dendrimers: From biomimicry to drug delivery and biomedical applications. Drug Discov. Today.

[144] Mendes L.P., Pan J., Torchilin V.P. (2017). Dendrimers as nanocarriers for nucleic acid and drug delivery in cancer therapy. Molecules.

[145] Cai H., Li K., Li J., Wen S., Chen Q., Shen M., Zheng L., Zhang G., Shi X. (2015). Dendrimer-Assisted Formation of Fe_3_O_4_/Au Nanocomposite Particles for Targeted Dual Mode CT/MR Imaging of Tumors. Small.

[146] Zheng Y., Fu F., Zhang M., Shen M., Zhu M., Shi X. (2014). Multifunctional dendrimers modified with alpha-tocopheryl succinate for targeted cancer therapy. Medchemcomm.

[147] Wiwattanapatapee R., Carreño-Gómez B., Malik N., Duncan R. (2000). Anionic PAMAM dendrimers rapidly cross adult rat intestine in vitro: A potential oral delivery system?. Pharm. Res..

[148] Zhu J., Fu F., Xiong Z., Shen M., Shi X. (2015). Dendrimer-entrapped gold nanoparticles modified with RGD peptide and alpha-tocopheryl succinate enable targeted theranostics of cancer cells. Colloids Surf. B. Biointerfaces.

[149] Madaan K., Kumar S., Poonia N., Lather V., Pandita D. (2014). Dendrimers in drug delivery and targeting: Drug-dendrimer interactions and toxicity issues. J. Pharm. Bioallied Sci..

[150] Liu Z., Jiang W., Nam J., Moon J.J., Kim B.Y.S. (2018). Immunomodulating Nanomedicine for Cancer Therapy. Nano Lett..

[151] Shetty Y., Prabhu P., Prabhakar B. (2018). Emerging vistas in theranostic medicine. Int. J. Pharm..

[152] Minchin R.F., Martin D.J. (2010). Minireview: Nanoparticles for Molecular Imaging—An Overview. Endocrinology.

[153] de Barros A., Tsourkas A., Saboury B., Cardoso V., Alavi A. (2012). Emerging role of radiolabeled nanoparticles as an effective diagnostic technique. EJNMMI Res..

[154] Liu Y., Welch M.J. (2012). Nanoparticles Labeled with Positron Emitting Nuclides: Advantages, Methods, and Applications. Bioconjug. Chem..

[155] Xing Y., Zhao J., Conti P.S., Chen K. (2014). Radiolabeled Nanoparticles for Multimodality Tumor Imaging. Theranostics.

[156] Kiessling F., Mertens M.E., Grimm J., Lammers T. (2014). Nanoparticles for Imaging: Top or Flop?. Radiology.

[157] Stockhofe K., Postema J., Schieferstein H., Ross T. (2014). Radiolabeling of Nanoparticles and Polymers for PET Imaging. Pharmaceuticals.

[158] Xing Y., Zhao J., Shi X., Conti P.S., Chen K. (2014). Recent Development of Radiolabeled Nanoparticles for PET Imaging. Austin J. Nanomed. Nanotechnol..

[159] Hong H., Zhang Y., Sun J., Cai W. (2009). Molecular imaging and therapy of cancer with radiolabeled nanoparticles. Nano Today.

[160] Phillips W.T., Goins B.A., Bao A. (2009). Radioactive liposomes. Wiley Interdiscip. Rev. Nanomed. Nanobiotechnol..

[161] Xie J., Lee S., Chen X. (2010). Nanoparticle-based theranostic agents. Adv. Drug Deliv. Rev..

[162] Su Y.-L., Hu S.-H. (2018). Functional Nanoparticles for Tumor Penetration of Therapeutics. Pharmaceutics.

[163] Sun Q., Ojha T., Kiessling F., Lammers T., Shi Y. (2017). Enhancing Tumor Penetration of Nanomedicines. Biomacromolecules.

[164] Cheng J., Teply B.A., Sherifi I., Sung J., Luther G., Gu F.X., Levy-Nissenbaum E., Radovic-Moreno A.F., Langer R., Farokhzad O.C. (2007). Formulation of functionalized PLGA-PEG nanoparticles for in vivo targeted drug delivery. Biomaterials.

[165] Farokhzad O.C., Cheng J., Teply B.A., Sherifi I., Jon S., Kantoff P.W., Richie J.P., Langer R. (2006). Targeted nanoparticle-aptamer bioconjugates for cancer chemotherapy in vivo. Proc. Natl. Acad. Sci. USA.

[166] Starpharma|Starpharma Commences Dendrimer-Docetaxel Clinical Trial Starpharma. https://starpharma.com/news/188.

[167] Tewari A. (2013). Prostate Cancer: A Comprehensive Perspective.

[168] Patel A.G., Kaufmann S.H. (2012). How does doxorubicin work?. Elife.

[169] Newling D.W.W. (1992). The use of adriamycin and its derivatives in the treatment of prostatic cancer. Cancer Chemother. Pharmacol..

[170] Matsumura Y., Hamaguchi T., Ura T., Muro K., Yamada Y., Shimada Y., Shirao K., Okusaka T., Ueno H., Ikeda M. (2004). Phase I clinical trial and pharmacokinetic evaluation of NK911, a micelle-encapsulated doxorubicin. Br. J. Cancer.

[171] Ki M.-H., Kim J.-E., Lee Y.-N., Noh S.M., An S.-W., Cho H.-J., Kim D.-D. (2014). Chitosan-based hybrid nanocomplex for siRNA delivery and its application for cancer therapy. Pharm. Res..

[172] Yu T., Liu X., Bolcato-Bellemin A.-L., Wang Y., Liu C., Erbacher P., Qu F., Rocchi P., Behr J.-P., Peng L. (2012). An amphiphilic dendrimer for effective delivery of small interfering RNA and gene silencing in vitro and in vivo. Angew. Chem. Int. Ed. Engl..

[173] Liu X.-X., Rocchi P., Qu F.-Q., Zheng S.-Q., Liang Z.-C., Gleave M., Iovanna J., Peng L. (2009). PAMAM dendrimers mediate siRNA delivery to target Hsp27 and produce potent antiproliferative effects on prostate cancer cells. ChemMedChem.

[174] Barthelemy P., Oumzil K., Rocchi P., Acunzo J. (2016). Hydrophobically Modified Antisense Oligonucleotides Comprising a Ketal Group.

